# Epitaxial Oxide Interfaces Create Poison‐Resistant CuO Sites for Environmental Catalysis

**DOI:** 10.1002/anie.7111765

**Published:** 2026-08-12

**Authors:** Lupeng Han, Yanqing Li, Yongjie Shen, Huijun Yu, Evangelina Pensa, Xuehui Yang, Yanqi Chen, Xiaonan Hu, Xiyang Wang, Song Li, Gaowu Qin, Wenqiang Qu, Ming Xie, Emiliano Cortés, Dengsong Zhang

**Affiliations:** ^1^ International Joint Laboratory of Catalytic Chemistry State Key Laboratory of Materials for Advanced Nuclear Energy Innovation Institute of Carbon Neutrality Department of Chemistry College of Sciences Shanghai University Shanghai People's Republic of China; ^2^ Institute For Chemical Reaction Design and Discovery (WPI‐ICReDD) Hokkaido University Sapporo Japan; ^3^ Nanoinstitute Munich Faculty of Physics Ludwig‐Maximilians‐Universität (LMU) Munich Germany; ^4^ Key Laboratory for Anisotropy and Texture of Materials (Ministry of Education) School of Materials Science and Engineering Northeastern University Shenyang People's Republic of China; ^5^ Department of Chemistry University of Toronto Toronto Ontario Canada; ^6^ Department of Chemical Engineering University of Bath Bath UK

**Keywords:** competitive adsorption, environmental catalysis, lattice oxygen, Lewis acid sites, oxide–oxide interface, SO_2_ resistance

## Abstract

Real exhaust streams rarely contain a single pollutant: NO_x_ coexists with volatile organic compounds (VOCs) in flue gas from petrochemical production, chemical manufacturing, and waste incineration, yet catalysts that couple NH_3_‐SCR with VOC oxidation typically suffer competitive adsorption, sulfur poisoning, and HCN byproduct formation. Here we engineer an epitaxially stabilized CuO overlayer on Ti_1‐x_In_x_O_2_ that breaks the activity–selectivity–stability constraint by creating electron‐poor, high‐symmetry Cu–O sites and activating lattice‐oxygen redox at the oxide–oxide interface. Interfacial strain and charge transfer increase Cu–O covalency and Lewis acidity, accelerating NO_x_ reduction via an Eley–Rideal pathway while diverting sulfate deposition away from Cu. Concurrently, interface‐activated lattice oxygen sustains deep oxidation of CH_3_SH (a representative S‐VOC) through a Mars–van Krevelen cycle, suppressing HCN. Epitaxial interfaces thus offer a general route to poison‐resistant multipollutant catalysis.

## Graphical abstract

An epitaxially stabilized CuO overlayer on Ti_1__−_ _x_ In _x_ O_2_ creates electron‐poor, high‐symmetry Cu–O sites that activate interfacial lattice‐oxygen redox. This interface promotes NO _x_ reduction through an Eley–Rideal pathway, diverts sulfate formation, and sustains CH_3_SH deep oxidation via a Mars–van Krevelen cycle, offering a route to poison‐resistant multipollutant catalysis.

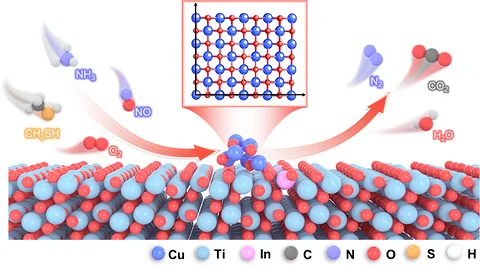

## 1 Introduction

Environmental catalysis processes, such as those for flue gas post‐treatment in petrochemical production, chemical manufacturing, and waste incineration, typically involve complex exhaust gases including nitrogen oxides (NO_x_) and volatile organic compounds (VOCs), which are pivotal to the formation of PM_2.5_ and O_3_ [1, 2, 3]. The synergistic catalytic elimination (SCE) of NO_x_ and VOCs not only protects the ecological environment but also significantly decreases operational costs for industry applications. The SCE process is fundamentally driven by coupled NH_3_‐selective catalytic reduction (NH_3_‐SCR) of NO_x_ and catalytic oxidation of VOCs [4]. Unlike single SCR or VOCs oxidation reaction, the coexistence of multiple gases reduces the SCE activity due to the strong competitive adsorption while generating toxic HCN from side reactions between oxygen‐containing intermediates and NH_3_ [5, 6]. Furthermore, the development of highly efficient and robust SCE catalysts poses substantial challenges due to the poisoning effects from the toxic by‐products of heteroatom‐containing VOCs [7, 8]. Typically, the catalytic oxidation of sulfur‐containing VOCs (S‐VOCs) generates SO_2_ and SO_4_^2^^−^ species [9, 10, 11]. Thermodynamically, the poisoning adsorption of SO_2_ and SO_4_^2^^−^ species blocks catalytic active sites and compromises redox properties of catalysts [12, 13, 14]. Therefore, the synergistic environmental catalysis necessitates optimal trade‐offs among activity, product selectivity, and sulfur tolerance.

The acidity and redox properties of catalysts play a crucial role in the catalytic reduction of NO_x_ and oxidation of VOCs. Acidic sites are responsible for the adsorption of NH_3_, while redox sites facilitate the oxidation of VOCs and activation of NO [15]. However, the oxidative capacity of catalysts is usually insufficient due to the strong competitive adsorption under complex environment, generating large amount of harmful HCN from the reaction between HCHO/HCOOH and NH_3_ [16]. It is an effective way to inhibit the HCN formation via boosting deep oxidation HCHO/HCOOH intermediates to CO_2_ at metal sites with strong oxidative capacity [17]. Unfortunately, the strong redox capacity at metal sites will further aggravate sulfur poisoning due to stronger adsorption of metal‐sulfur bond during the oxidation of S‐VOCs. Over the past decades, extensive efforts have been devoted to improving the sulfur tolerance of heterogeneous catalysts. Typically, CeO_2_, MgO, Fe_2_O_3_ as sacrificial sites or encapsulation structures were decorated on the catalyst surface to alleviate the sulfation of active sites, but this approach often blocks access to the catalytically active sites [18, 19, 20, 21]. Another strategy is to increase the surface acidity of catalysts by modifying SiO_2_ and SO_4_^2−^ to inhibit the adsorption of SO_2_/SO_4_^2−^ species, but inevitably decreasing the oxidative capacity of metal sites [22, 23, 24]. To date, the catalyst design for simultaneously achieving sulfur resistance and high oxidative capacity remains a critical challenge for efficient and stable environmental catalysis.

Herein, we designed and synthesized an epitaxially stabilized CuO overlayer on a Ti_1‐x_In_x_O_2_ solid solution. The lattice matching‐induced interface strains between CuO and Ti_1‐x_In_x_O_2_ not only stabilize the symmetric CuO_4_ planar square configuration but also promote efficient electron transfer from CuO to Ti_1‐x_In_x_O_2_ support. These regulations decrease the electron density of Cu 3d orbitals and efficiently activate lattice oxygen as new redox sites to substitute traditional metal sites easily poisoned by sulfur. Therefore, it enhances both the Lewis acidity of Cu–O sites and the oxidative capacity of catalysts. The tailored catalyst successfully breaks activity‐selectivity‐stability trade‐off for the SCE of NO_x_ and CH_3_SH (most representative molecule of S‐VOCs), delivering high NO_x_/CH_3_SH conversion with good N_2_/CO_2_ selectivity while maintaining superior stability during the long‐term tests. The efficient and stable environmental catalysis process involves two synergistic catalytic cycle, where the SCR reaction proceeds at Cu–O sites with strong Lewis acidity via the Eley–Rideal (E–R) mechanism and the deep oxidation of S‐VOCs achieves at the activated lattice oxygen sites via the Mars–van Krevelen (MvK) mechanism.

## 2 Results and Discussion

### 2.1 Geometry and Electronic Structures of Epitaxial CuO Catalysts

The Ti_1‐x_In_x_O_2_ solid solutions and CuO/Ti_1‐x_In_x_O_2_ catalysts were prepared by sol–gel and wet impregnation methods, respectively. The transmission electron microscopy‐energy dispersive x‐ray spectroscopy (TEM‐EDS) mapping and Raman spectra indicate the formation of Ti_1‐x_In_x_O_2_ solid solutions (Figures S1 and S2). The Rietveld refined x‐ray powder diffraction (XRD) of CuO/Ti_1‐x_In_x_O_2_ illustrates that In ions are incorporated into the TiO_2_ lattice (Figure 1a) [25]. To observe the epitaxial interface and atomic structure of CuO/Ti_1‐x_In_x_O_2_, the aberration‐corrected high‐angle annular dark‐field scanning transmission electron microscopy (HAADF‐STEM) and bright‐field scanning transmission electron microscopy (BF‐STEM) images were measured and simulated, respectively. The atomic‐scale BF‐STEM image demonstrates the epitaxial growth of CuO overlayer on the Ti_1‐x_In_x_O_2_ support (Figure 1b). The doping of larger In^3+^ ions leads to the tensile strain of Ti_1‐x_In_x_O_2_ (003) from standard 3.21 Å to 3.37 Å while CuO displays the small tensile strain in (010) facet from 3.41 to 3.42 Å due to epitaxial matching (Figures 1b and S3) [26]. In doping in TiO_2_ also results in the tensile strain of Ti_1‐x_In_x_O_2_ (006) facet from 1.58 to 1.65 Å (Figure S4). The Rietveld‐refined XRD and Ti K‐edge extended x‐ray absorption fine structure (EXAFS) analysis further demonstrates the In‐doping induced lattice expansion of TiO_2_ (Tables S1–S3 and Figure S5). This distortion alters the coordination geometry around Cu atoms, leading to the observed bond angle change. The image simulations of epitaxial CuO (Figure 1c,d) reveal an unusual symmetric coordination environment distinct from bulk CuO, characterized by a transition of the Cu─O─Cu bond angle from ∼82° (theoretical value: 84°) to −90°(Figures S6 and S7) [27]. Along the [010] zone axis, the measured lattice spacings of epitaxial CuO are (100) plane with compressive 4.53 Å (standard 4.59 Å) and CuO (001) plane with tensile 5.16 Å (standard 5.04 Å) (Figure S6). Above results confirm that the epitaxial CuO‐Ti_1‐x_In_x_O_2_ interface stabilizes high‐symmetry Cu–O sites by lattice matching‐induced interface strains.

**FIGURE 1:**
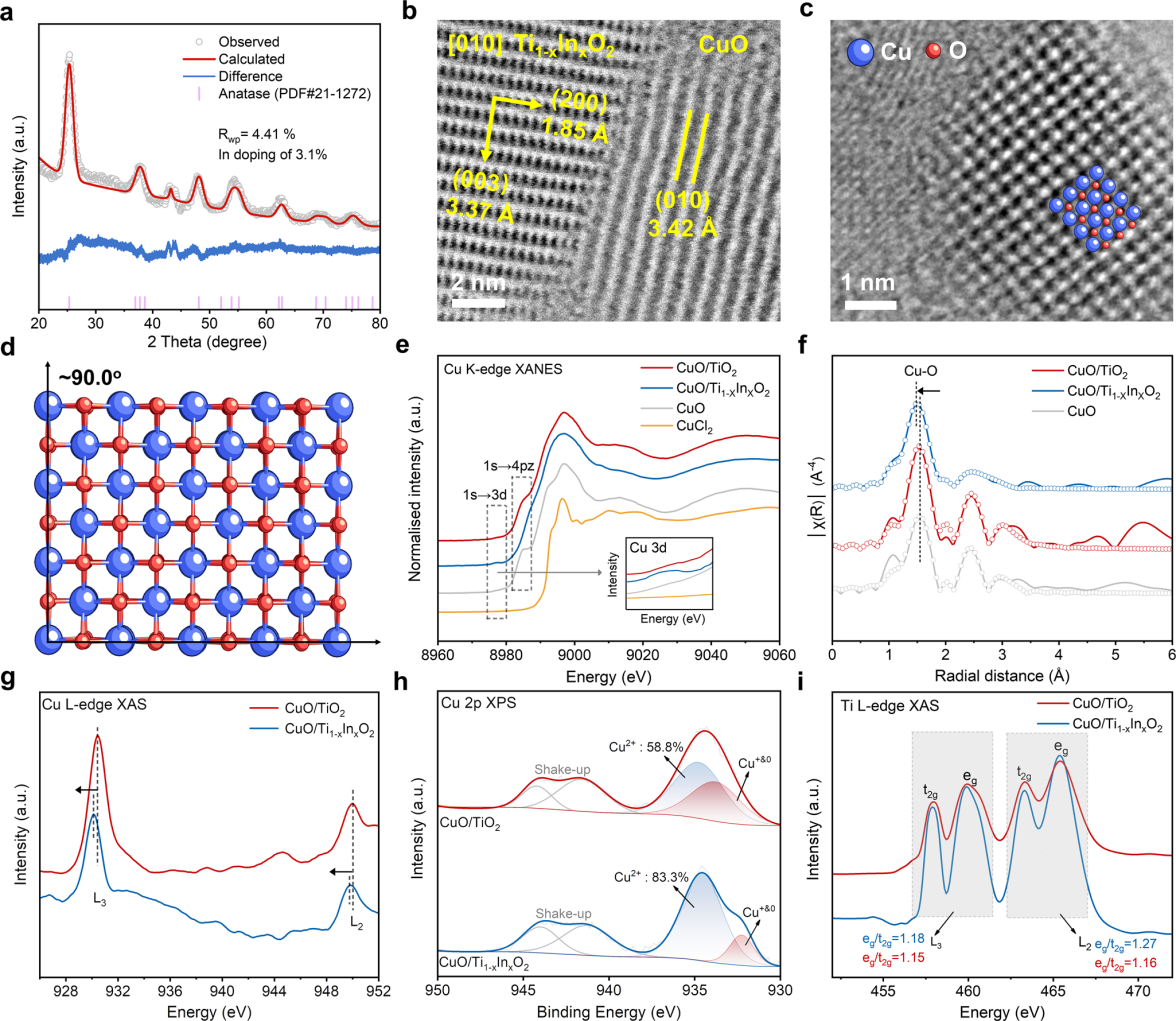
Geometry and electronic structures of catalysts. (a) The Rietveld XRD pattern of CuO/Ti_1‐x_In_x_O_2_. BF‐STEM (b) and HAADF‐STEM (c) images of CuO/Ti_1‐x_In_x_O_2_. (d) The image simulation of CuO in CuO/Ti_1‐x_In_x_O_2_. (e) The Cu K‐edge XANES spectra of catalysts and the inset is the enlarged pre‐edge peak of Cu 1s → 3d. (f) FT‐EXAFS fitting curves for CuO/TiO_2_ and CuO/Ti_1‐x_In_x_O_2_ in R space. (g) The Cu L‐edge XAS spectra of CuO/TiO_2_ and CuO/Ti_1‐x_In_x_O_2_. (h) Cu 2p XPS spectra of CuO/TiO_2_ and CuO/Ti_1‐x_In_x_O_2_. (i) Ti L‐edge XAS spectra of CuO/TiO_2_ and CuO/Ti_1‐x_In_x_O_2_.

To confirm the coordination environment and electronic structure of CuO at CuO‐Ti_1‐x_In_x_O_2_ interface, Cu K‐edge x‐ray absorption near‐edge structure (XANES) and EXAFS spectra were tested, respectively. Unlike standard CuO and CuO/TiO_2_ samples, the CuO/Ti_1‐x_In_x_O_2_ exhibits the disappearance of the Cu 1s → 4pz peak and a continuum in the Cu 1s → 4p region in Cu K‐edge XANES spectra (Figure 1e). These features arise from a high‐symmetry CuO_4_ planar square, which induces an increased number of vacant Cu 3d orbitals, enhanced Cu─O bond covalency, and strengthened Cu 3d‐4p interactions. Such a coordination symmetry is close with CuCl_4_ planar square field of standard CuCl_2_ (Figure 1e). Moreover, CuO/Ti_1‐x_In_x_O_2_ shows an additional pre‐edge peak (Cu 1s → 3d), which is attributed to the electron transfer from Cu 3d orbitals to the Ti_1‐x_In_x_O_2_ support driven by the unique epitaxial interface. Such a charge transfer induces obvious p–d orbital hybridization, endowing Cu 3d orbitals with partial p‐orbital character. This effect partially relaxes the dipole‐forbidden limitation of the 1s→3d transition, thereby generating a weak pre‐edge peak. Figure 1f shows well‐fitted EXAFS spectra using theory model structure, displaying the peak around 1.55 Å assigned to the Cu‐O (first shell) and 2.45 Å assigned to Cu─O─Cu/Ti (second shell) of CuO/Ti_1‐x_In_x_O_2_ (Figure S8). Compared with standard CuO (Cu─O bond length: 1.96 Å) and CuO/TiO_2_ (1.96 Å), the Cu─O bond length in CuO/Ti_1‐x_In_x_O_2_ (1.94 Å) decreases, and the Cu─O coordination numbers obviously increase from 3.89 to 3.99 (Table S4). The EXAFS results about bond length and coordination reflect the improvement of Cu─O geometry symmetry and the enhancement of Cu─O covalency. In addition, the peak intensity of Cu─O─Cu/Ti shell in CuO/Ti_1‐x_In_x_O_2_ has a distinct reduction compared to CuO/TiO_2_, suggesting the higher dispersed state of Cu species on the support surface. Moreover, the peak positions in Cu L‐edge XAS spectra of CuO/Ti_1‐x_In_x_O_2_ have a shift to lower energy compared with CuO/TiO_2_ (Figure 1g), indicating that the elevated Cu─O symmetry lowers the energy of the Cu 3d orbitals due to the enhanced Cu─O covalency and strong interface electron transfers. The x‐ray photoelectron spectroscopy (XPS) of Cu 2p shows a higher average valence state of Cu atom in CuO/Ti_1‐x_In_x_O_2_ than CuO/TiO_2_ for the higher Cu^2+^ fraction (83.3% vs. 58.8%, Figure 1h). The density functional theory (DFT) calculations were performed to clarify the interface electronic states of CuO/Ti_1‐x_In_x_O_2_ and CuO/TiO_2_ based on the structural models derived from XRD and TEM results (Figure S9). The charge density difference and Bader charge analysis indicate a significantly different charge distribution with 0.53 e transfer from CuO to Ti_1‐x_In_x_O_2_ but reverse 1.01 e from TiO_2_ to CuO (Figure S10). The average electron loss (−0.94 e) per Cu atom in CuO/Ti_1‐x_In_x_O_2_ is more than that (0.67 e) in CuO/TiO_2_ (Table S5). The Ti L‐edge XAS spectra also manifest the more electron‐rich state in Ti 3d t_2g_ orbital because of the increased peak intensity ratios of e_g_/t_2g_ in CuO/Ti_1‐x_In_x_O_2_ compared with CuO/TiO_2_ (Figure 1i). Above results demonstrate that CuO/Ti_1‐x_In_x_O_2_ possesses high‐symmetry electron‐poor Cu─O sites distinctly different from CuO/TiO_2_.

### 2.2 Strongly Lewis Acid and Activated Lattice Oxygen Sites

CuO usually acts as a Lewis base due to the relatively fully filled electrons in the Cu 3d orbitals and the presence of lone pairs on the O^2−^ ions. The enhancement of covalency and geometric symmetry promotes the formation and stabilization of electron‐poor Cu ions, which act as efficient Lewis acid sites. To demonstrate the Lewis acidity of engineered Cu─O sites, pyridine adsorption infrared spectroscopy (Py‐IR) was employed. As shown in Figure 2a, the bands at 1552 cm^−1^ originate from pyridine adsorption on Brønsted acid sites, while the bands at 1603, 1475, and 1442 cm^−1^ represent the pyridine adsorption on Lewis acid sites [28, 29, 30]. Clearly, CuO/Ti_1‐x_In_x_O_2_ has much more Lewis acid sites than CuO/TiO_2_ as well as TiInO_x_ and TiO_2_ support, demonstrating the strong Lewis acidity of CuO_x_ sites in CuO/Ti_1‐x_In_x_O_2_ (Figure S11). Besides, NH_3_ temperature‐programmed desorption with mass spectrum (NH_3_‐TPD‐MS) was used to investigate the acidic features of catalysts. As seen in Figure 2b, the desorption peaks below 250°C correspond to weak acid sites, the ones at 250°C–500°C are assigned to medium strong acid sites, and the ones above 500°C are attributed to strong acid sites [31]. Clearly, CuO/Ti_1‐x_In_x_O_2_ shows much more acid sites than CuO/TiO_2_ as well as Ti_1‐x_In_x_O_2_ and TiO_2_ supports (Figure S12 and Table S6). The stronger acidity is originated from the Lewis acidic CuO_x_ sites in CuO/Ti_1‐x_In_x_O_2_, which plays significant roles in SCE of NO_x_ and CH_3_SH. Correspondingly, CuO/Ti_1‐x_In_x_O_2_ possesses more weak basic sites and less strong basic sites compared to CuO/TiO_2_ from the CO_2_ temperature‐programmed desorption with mass spectrum (CO_2_‐TPD‐MS, Figure 2c). The redox properties of CuO/TiO_2_ and CuO/Ti_1‐x_In_x_O_2_ were then probed by H_2_ temperature‐programmed reduction (H_2_‐TPR). As shown in Figure 2d, both catalysts show two hydrogen reduction peaks attributed to the stepwise reduction of Cu^2^^+^→Cu^+^ and Cu^+^→Cu^0^ with the increasing temperature, respectively [32]. Compared to CuO/TiO_2_, CuO/Ti_1‐x_In_x_O_2_ exhibits lower onset, sharp, and symmetrical reduction peaks below 200°C, which suggests the presence of more uniform and highly reducible CuO_x_ species. It can be inferred that the enhanced geometric symmetry and increased covalency of the Cu─O bonds synergistically weaken the Cu─O bonding and activate the surface lattice oxygen, thereby markedly enhancing its reducibility. Moreover, the O 1s XPS spectra of CuO/Ti_1‐x_In_x_O_2_ extra present activated lattice oxygen species at 531.0 eV compared to CuO/TiO_2_, Ti_1‐x_In_x_O_2_ and TiO_2_ (Figures 2e and S13), which will facilitate redox property of lattice oxygen sites in catalytic oxidation reactions [33]. In O K‐edge XANES spectra (Figure 2f), the high‐energy shift of peak a in Ti_1‐x_In_x_O_2_ relative to TiO_2_ indicates enhanced metal–oxygen covalency due to In doping, while its slight low‐energy shift in CuO/Ti_1‐x_In_x_O_2_ suggests reduced covalency due to electron transfer from CuO to the Ti_1‐x_In_x_O_2_ support. These results, together with O 1s XPS, demonstrate that In doping and the epitaxial interface synergistically activate lattice oxygen species [34]. The electron paramagnetic resonance (EPR) spectrum suggests that CuO/Ti_1‐x_In_x_O_2_ possesses an increased oxygen vacancies compared to CuO/TiO_2_, Ti_1‐x_In_x_O_2_ and TiO_2_ (Figures 2g and S14). The DFT calculations shows that CuO_x_ sites of CuO/Ti_1‐x_In_x_O_2_ shows smaller formation energy (1.24 eV) of O vacancy compared to CuO/TiO_2_ (2.80 eV), which promotes the participation of lattice oxygen in the reaction (Figure 2h). Furthermore, CuO/Ti_1‐x_In_x_O_2_ shows more negative O_2_ adsorption energy than CuO/TiO_2_ (−0.31 vs. −0.20 eV, Figure 2i), suggesting CuO/Ti_1‐x_In_x_O_2_ is more favorable for O_2_ adsorption and activation. Based on above results, the CuO/Ti_1‐x_In_x_O_2_ catalyst featuring Lewis acidic Cu and activated lattice oxygen sites is anticipated to enhance the activity and stability of SCE of NO_x_ and sulfur‐containing VOCs.

**FIGURE 2:**
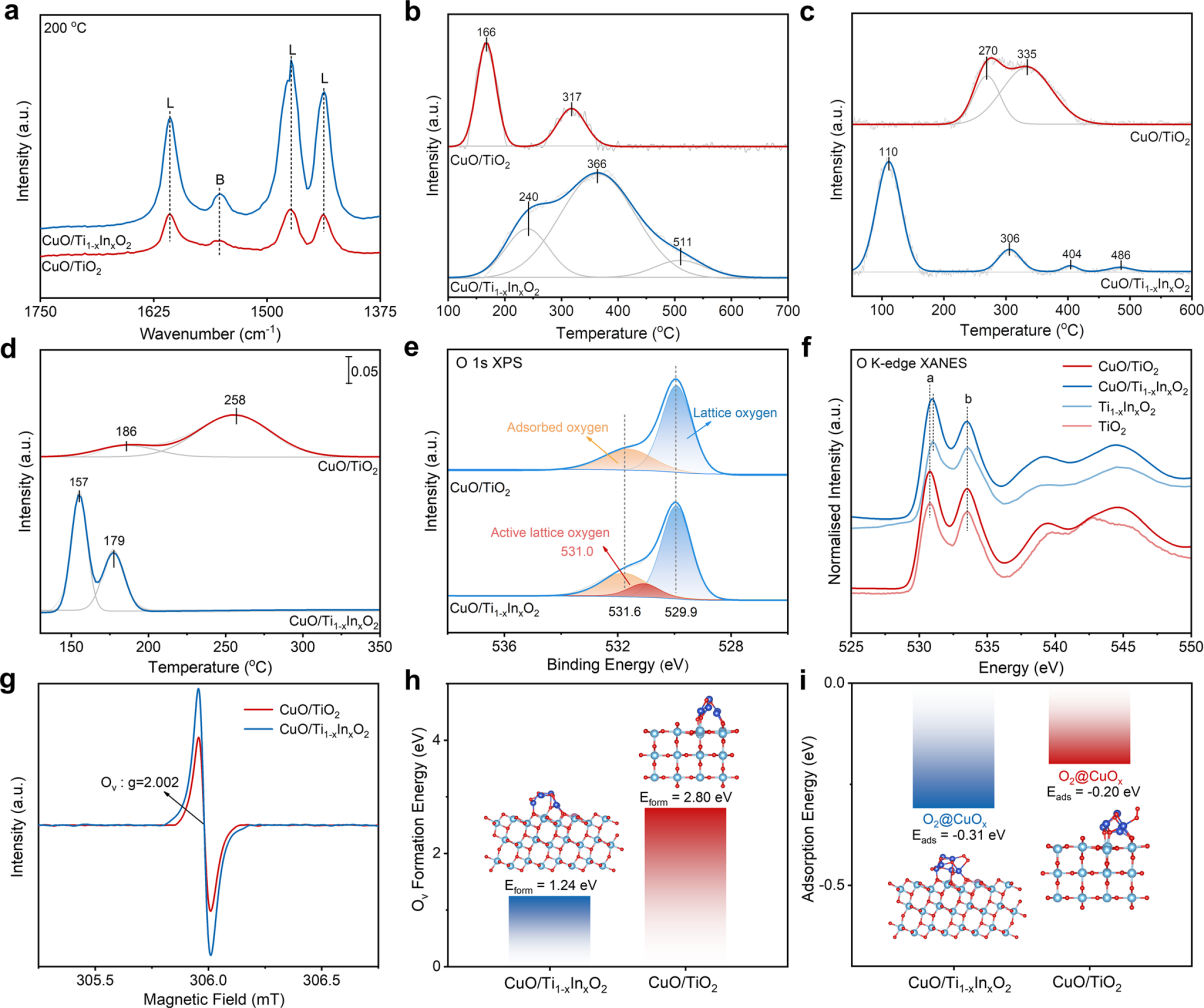
Lewis acidity and lattice oxygen sites of catalysts. (a) Py‐IR profiles of CuO/TiO_2_ and CuO/Ti_1‐x_In_x_O_2_ at 200°C. (b) NH_3_‐TPD‐MS profiles (*m*/*z* = 17) of CuO/TiO_2_ and CuO/Ti_1‐x_In_x_O_2_. (c) CO_2_ ‐TPD profiles (*m*/*z* = 44) of CuO/TiO_2_ and CuO/Ti_1‐x_In_x_O_2_. (d) H_2_‐TPR profiles of CuO/TiO_2_ and CuO/Ti_1‐x_In_x_O_2_. (e) O 1s XPS spectra of CuO/TiO_2_ and CuO/Ti_1‐x_In_x_O_2_. (f) The O K‐edge XAS spectra of TiO_2_, Ti_1‐x_In_x_O_2_, CuO/TiO_2_ and CuO/Ti_1‐x_In_x_O_2_. (g) EPR spectra of CuO/TiO_2_ and CuO/Ti_1‐x_In_x_O_2_. (h) DFT calculated oxygen vacancy formation energy on CuO/TiO_2_ and CuO/Ti_1‐x_In_x_O_2_ models. (i) DFT calculated O_2_ adsorption energy on CuO/TiO_2_ and CuO/Ti_1‐x_In_x_O_2_ models.

### 2.3 The Synergistic Environmental Catalytic Performance

As CH_3_SH is the most representative odorous molecule among sulfur‐containing VOCs, the synergistic elimination performances of NO_x_ and CH_3_SH were investigated (Figure 3a,b). For CuO/Ti_1‐x_In_x_O_2_, the CH_3_SH conversion approaches 100% and the NO_x_ conversion is higher than 85% in the temperature range of 270°C–360°C. Notably, the CO_2_ selectivity can be maintained above 90% and N_2_ selectivity keeps above 80%. As a comparison, CH_3_SH/NO_x_ conversion and CO_2_/N_2_ selectivity of CuO/TiO_2_ are all significantly inferior to CuO/Ti_1‐x_In_x_O_2_. In addition, CuO/Ti_1‐ x_In_x_O_2_ hardly produces CO and HCN in the temperature range of 180°C–360°C, while both are readily generated over CuO/TiO_2_ (Figure S15). In order to probe the effect of CH_3_SH on NH_3_‐SCR performance, the NH_3_‐SCR activity of CuO/Ti_1‐x_In_x_O_2_ and CuO/TiO_2_ was also investigated in the absence of CH_3_SH (Figure S16). Both CuO/Ti_1‐x_In_x_O_2_ and CuO/TiO_2_ show better low‐temperature activity in the absence of CH_3_SH while exhibiting higher high‐temperature activity in the presence of CH_3_SH. This result indicates that the sulfation of active sites decreases the redox properties but increase the acidic properties of catalysts [35]. Compared to CuO/TiO_2_, CuO/Ti_1‐x_In_x_O_2_ exhibits lower apparent activation energy of NH_3_‐SCR (38.3 vs. 47.0 kJ/mol) and CH_3_SH oxidation (14.9 vs. 25.6 kJ/mol) (Figures S17 and S18), which indicates higher intrinsic activity of NH_3_‐SCR and CH_3_SH oxidation over CuO/Ti_1‐x_In_x_O_2_. CuO/Ti_1‐x_In_x_O_2_ can maintain NO_x_ and CH_3_SH conversions more than 90% for 20 h at 300°C under GHSV of 25,000 h^−1^ (Figure S19). To further evaluate the catalytic stability under kinetic control, the deactivation behavior was investigated at 300°C with low NO_x_ and CH_3_SH conversions under a high GHSV of 500,000 h^−1^ (Figure 3c). The experimental data were quantitatively analyzed using an exponential decay model: $y=A\cdot e^{-K_{d}\cdot x}+y_{0}$+ y_0_, where y represents the conversion, A is the initial amplitude, K_d_ is the deactivation rate constant, x is the time, and y_0_ is the asymptotic conversion. As show in Figure 3c, the fitted curves are in good agreement with the experimental data, with *R* ^2^ values exceeding 0.90, indicating the high reliability of the fitting (Table S7). Based on the fitting results (Table S7), the K_d_ values for both NO_x_ and CH_3_SH conversions over CuO/Ti_1‐x_In_x_O_2_ are substantially lower than those over CuO/TiO_2_, demonstrating the superior catalytic stability of CuO/Ti_1‐x_In_x_O_2_. The above results demonstrate that CuO/Ti_1‐x_In_x_O_2_ exhibits superior SCE activity, N_2_/CO_2_ selectivity and catalytic stability compared to CuO/TiO_2_.

**FIGURE 3:**
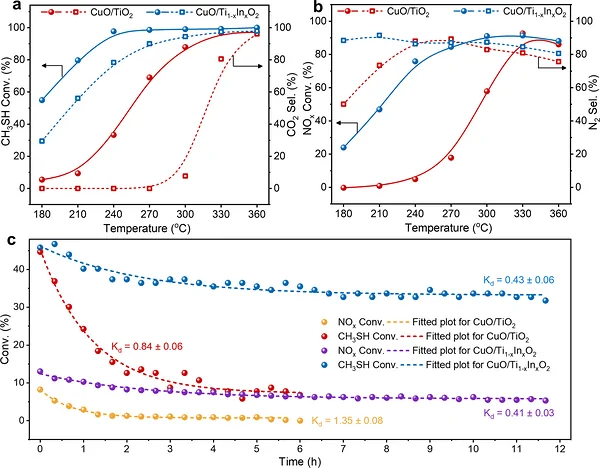
The synergistic catalytic performance. Plots of CH_3_SH conversion and CO_2_ selectivity (a) as well as NO_x_ conversion and N_2_ selectivity (b) as a function of reaction temperature over CuO/TiO_2_ and CuO/Ti_1‐x_In_x_O_2_ for the synergistic catalytic elimination of NO_x_ and CH_3_SH under the reaction conditions: 500 ppm NO, 500 ppm NH_3_, 100 ppm CH_3_SH, 5 vol% O_2_, N_2_ as the balance gas, and GHSV of 25,000 h^−1^. (c) Plots of CH_3_SH conversion and NO_x_ conversion as a function of reaction time over CuO/TiO_2_ and CuO/Ti_1‐x_In_x_O_2_ under the reaction conditions: 300°C, 500 ppm NO, 500 ppm NH_3_, 100 ppm CH_3_SH, 5 vol% O_2_, N_2_ as the balance gas, and GHSV of 500,000 h^−1^. The dashed lines represent the fitted curves obtained from the exponential decay model.

### 2.4 Synergistic Catalytic Reaction Mechanism

In order to clarify the nature for synergistic catalytic performance of CuO/Ti_1‐x_In_x_O_2_, the dynamic behaviors of adsorbed species were investigated under different reaction conditions. Both NO+O_2_‐TPD and CH_3_SH+O_2_‐TPD profiles indicate that CuO/Ti_1‐x_In_x_O_2_ exhibits enhanced adsorption quantity and adsorption strength of NO and CH_3_SH compared to CuO/TiO_2_ (Figures S20 and S21) [36]. DFT calculations indicate that the adsorption energies of NO, NH_3_ and CH_3_SH on the Cu sites of CuO/Ti_1‐x_In_x_O_2_ are more negative than those on CuO/TiO_2_ (NO: −0.52 vs. −0.16 eV; NH_3_: −1.41 vs. −1.30 eV; CH_3_SH: −1.18 vs. −1.00 eV), further suggesting the more favorable adsorption and activation of reactants (Figure S22–S24). As shown in Figure 4a,b, in situ diffuse reflectance infrared Fourier transform (DRIFT) spectra of CH_3_SH+O_2_ adsorption manifest that the HCOO^−^ (1582 cm^−1^), CO_3_^2−^ (1418 and 1300 cm^−1^), CH_3_S (1338 cm^−1^), CH_2_SH (1252 cm^−1^), and SO_4_^2−^ (1137 cm^−1^) species emerge over both catalysts [37, 38, 39, 40, 41, 42, 43]. The in situ DRIFT spectra of the transient reactions between O_2_ and pre‐adsorbed CH_3_SH also show the same intermediates (Figure S25). Therefore, the CH_3_SH oxidation pathway is proposed as follows *CH_3_SH→*CH_2_S→*HCOO^−^→*CO_3_^2−^→CO_2_. Notably, CuO/Ti_1‐x_In_x_O_2_ shows much more CO_3_^2−^ species compared to CuO/TiO_2_, indicating that the strong oxidative capacity boosts the deep oxidation of CH_3_SH to CO_3_^2−^ species that further decompose to CO_2_. The enhanced oxidative capacity of CuO/Ti_1‐x_In_x_O_2_ arises from more activated lattice oxygen sites endowed with favorable oxygen vacancy generation and O_2_ adsorption. This synergistic effect accelerates the reaction kinetics of CH_3_SH oxidation via the MvK mechanism [44]. However, the band intensity of SO_4_^2−^ species over CuO/Ti_1‐x_In_x_O_2_ is stronger than CuO/TiO_2_. The superior catalytic stability of CuO/Ti_1‐x_In_x_O_2_ indicates that the Lewis acidic Cu–O sites are not occupied by SO_4_^2−^ species and thus remain the original catalytic activity for SCR and CH_3_SH oxidation. It is inferred that SO_2_ as an acidic gas is not prone to adsorb on the Lewis acidic Cu–O sites. Cu 2p XPS, Cu K‐edge XANES, and EXAFS results (Figures S26–S28) indeed evidence the less changes observed in the valence state and coordination structure of Cu in S‐CuO/Ti_1‐x_In_x_O_2_ relative to S‐CuO/TiO_2_ (S‐catalysts denotes those after 20 h SCE tests). In order to further uncover the bonding sites of SO_4_^2−^ species on S‐CuO/Ti_1‐x_In_x_O_2_ and S‐CuO/TiO_2_, the element line scanning of TEM‐EDS image was conducted (Figures 4c and S29). For S‐CuO/TiO_2_, the variation trends of S and Cu elements are consistent, indicating that SO_4_^2−^ species predominantly deposit on CuO. As for S‐CuO/Ti_1‐x_In_x_O_2_, the variation trends of S and In elements are more similar, indicating that SO_4_^2−^ species combines with the In sites of Ti_1‐x_In_x_O_2_ support. The In 3d XPS spectra of S‐CuO/Ti_1‐x_In_x_O_2_ shows a shift to higher binding energy compared to CuO/Ti_1‐x_In_x_O_2_ (Figure S30), confirming the significant change of electron structure for the bonding of SO_4_^2−^ with In sites. DFT calculations also suggest that SO_2_ and SO_4_^2−^ adsorption are more favorable on In sites (with more negative adsorption energies) than on Cu sites of CuO/Ti_1‐x_InxO_2_ (Figure 4d,e). Furthermore, in situ DRIFT spectra of the transient reactions between NH_3_ and NO+O_2_ were also investigated over CH_3_SH+O_2_ pre‐treated catalysts. As shown in Figure 4f,g, the NH_3_ species (1616, 1264, and 1143 cm^−1^) are detected after NH_3_ adsorption [45, 46, 47], and the NH_3_ adsorption amount of CuO/Ti_1‐x_In_x_O_2_ is more than that on CuO/TiO_2_. The NH_3_ species are gradually consumed without emerging nitrogen oxide species after introducing NO+O_2_, indicating the typical E‐R mechanism where adsorbed NH_3_ directly reacts with gaseous NO_x_ on the Cu active sites [48]. It is notable that the consumption rate of NH_3_ species over CuO/Ti_1‐x_In_x_O_2_ is much faster than CuO/TiO_2_, indicating the higher reactivity of NH_3_ species. As shown in Figure 4h,i, a small amount of nitrocompounds (Cu‐NO_2_) species (1348, 1327, and 1188 cm^−1^) present on the surface of CuO/TiO_2_ and CuO/Ti_1‐x_In_x_O_2_ after the adsorption of NO+O_2_ [49, 50]. Subsequently, Cu‐NO_2_ species are rapidly consumed after introducing NH_3_, and meanwhile a large amount of NH_3_ species (1616, 1530, 1264, and 1143 cm^−1^) appear on the surface [45, 46, 47]. Notably, the adsorption of NH_3_ species on CuO/Ti_1‐x_In_x_O_2_ is significantly stronger than that on CuO/TiO_2_. These results further suggest that CuO/Ti_1‐x_In_x_O_2_ mainly proceed SCR reaction via E–R reaction pathways where the adsorbed NH_3_ species are more reactive over CuO/Ti_1‐x_In_x_O_2_.

**FIGURE 4:**
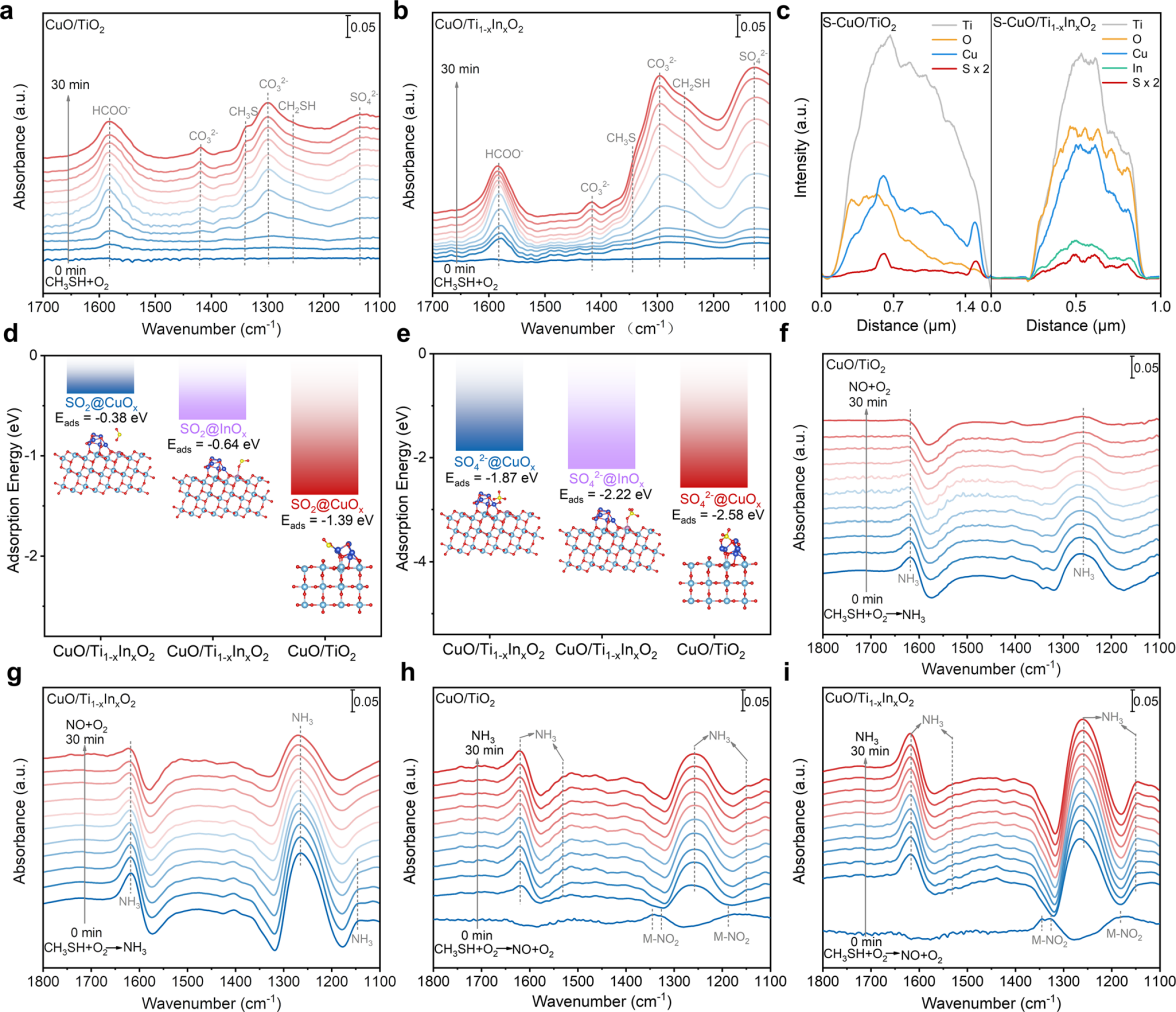
Reaction mechanism. In situ DRIFT spectra of CH_3_SH+O_2_ adsorption over CuO/TiO_2_ (a) and CuO/Ti_1‐x_In_x_O_2_ (b) for 30 min at 240°C. (c) The element line scanning of TEM‐EDS of CuO/TiO_2_ and CuO/Ti_1‐x_In_x_O_2_. SO_2_ (d) and SO_4_^2−^(e) adsorption energy of CuO/TiO_2_ and CuO/Ti_1‐x_In_x_O_2_. In situ DRIFT spectra of the transient reactions between NO+O_2_ and pre‐adsorbed NH_3_ at 240°C as a function of time over CuO/TiO_2_ (f) and CuO/Ti_1‐x_In_x_O_2_ (g). In situ DRIFT spectra of the transient reactions and between NH_3_ and pre‐adsorbed NO+O_2_ at 240°C as a function of time over CuO/TiO_2_ (h) and CuO/Ti_1‐x_In_x_O_2_ (i).

Based on in situ DRIFT spectra of CuO/Ti_1‐x_In_x_O_2_ and CuO/TiO_2_, the reaction pathway of SCE of NO_x_ and CH_3_SH is proposed involving two circles of CH_3_SH oxidation and NO_x_ reduction reactions. As illustrated in Figure 5 (Figure S31; Tables S8 and S9), the synergistic catalytic reaction begins with CH_3_SH adsorption followed by the reaction of *CH_3_SH with 1/4 O_2_ to form *CH_2_SH and 1/2 H_2_O. The *CH_2_SH subsequently reacts with 1/2 O_2_ to form *H_2_CO and *SH, in which *SH preferentially adsorbs on Cu sites rather than In sites on CuO/Ti_1‐x_In_x_O_2_ (Figure S32). In the next step, *H_2_CO and *SH react with 2 O_2_ to form *HCOO, *SO_2_ and H_2_O, where *SO_2_ is more prone to adsorb on In sites rather than Cu sites of CuO/Ti_1‐x_In_x_O_2_ (Figure S32). Whereas, *HCOO and *SO_2_ both adsorb on Cu sites of CuO/TiO_2_. And then *HCOO and *SO_2_ react with 3/2 O_2_ to form *CO_3_, *SO_4_ and H_2_O. The CH_3_SH oxidation reaction circle completes with decomposition of *CO_3_ to CO_2_ leaving *H, *O and *SO_4_. Notably, *SO_4_ adsorbs on In sites of CuO/Ti_1‐x_In_x_O_2_ but on Cu sites of CuO/TiO_2_. Afterwards, the SCR cycle begins with NH_3_ adsorption, followed by the reaction of *NH_3_ with *O to form *NH_2_ and H_2_O. The *NH_2_ then reacts with gaseous NO, generating *NH_2_NO, which finally decomposes into N_2_ and H_2_O, completing the SCR cycle. Due to the preferential adsorption of *SO_4_ on In sites, the Cu sites in CuO/Ti_1‐x_In_x_O_2_ remain available for successive CH_3_SH oxidation and SCR reactions, enabling efficient SCE of NO_x_ and CH_3_SH. In contrast, *SO_4_ adsorption on Cu sites in CuO/TiO_2_ leads to gradual catalyst poisoning and deactivation of the SCE reaction. Free energy diagrams of the two catalysts reveal two distinct energy barriers in the oxidative dehydrogenation steps of CH_3_SH and NH_3_ (Figures 5, S31, and S33), where CuO/Ti_1‐x_In_x_O_2_ exhibits lower energy barriers than CuO/TiO_2_ (0.12 vs. 0.57 eV and 0.31 vs. 0.89 eV, respectively). These theoretical results indicate that the SCE of NO_x_ and CH_3_SH is thermodynamically more favorable over CuO/Ti_1‐x_In_x_O_2_.

**FIGURE 5:**
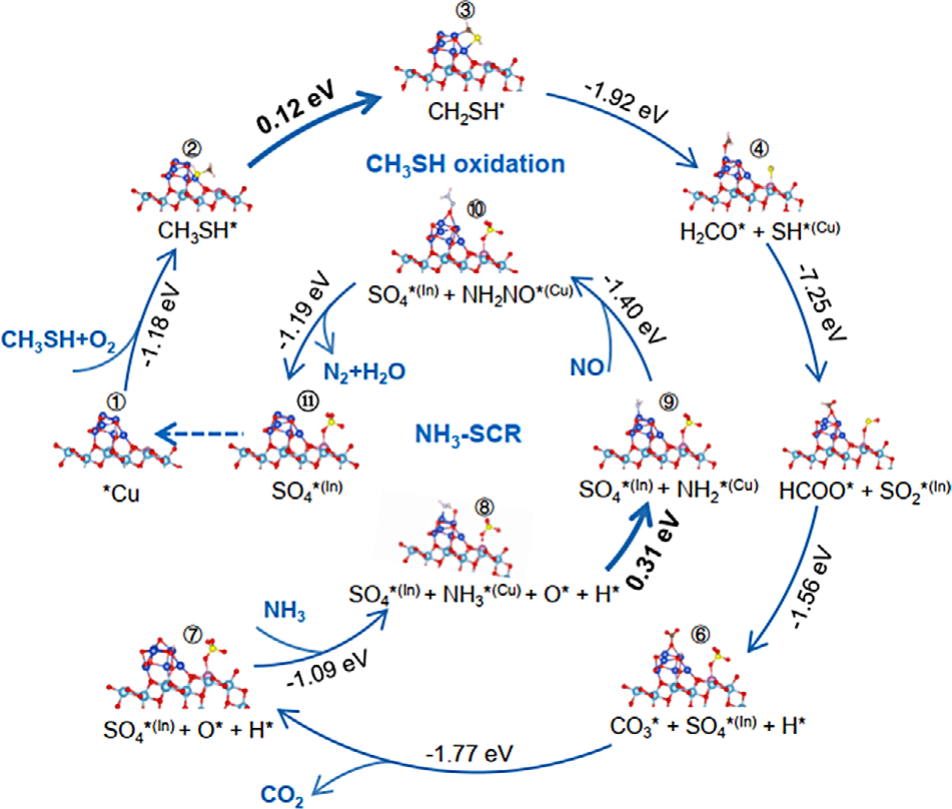
Reaction energy diagram for CH_3_SH oxidation and NH_3_‐SCR over CuO/Ti_1‐x_In_x_O_2_.

## 3 Conclusion

In summary, we successfully engineered an epitaxially stabilized CuO overlayer on a Ti_1‐x_In_x_O_2_ support, which owns a high‐symmetry electron‐deficient Cu─O sites coupled with activated lattice oxygen at the CuO/Ti_1__−__x_In_x_O_2_ interface, which exhibits exceptional activity, sulfur resistance, and N_2_/CO_2_ selectivity for the SCE of NO_x_ and CH_3_SH. The epitaxial interfacial strain stabilizes a near‐ideal CuO_4_ planar square configuration and facilitates efficient electron transfer from CuO to the Ti_1‐x_In_x_O_2_ support. The strongly Lewis‐acidic Cu─O sites enhance the NH_3_ adsorption/activation while transferring the SO_2_ adsorption/sulfate deposition on neighboring In sites, which enables sulfur‐tolerant SCR reaction via the E–R mechanism. Meanwhile, the activated lattice oxygen sites boost oxygen vacancy generation and O_2_ adsorption, triggering the deep oxidation of CH_3_SH through the MvK pathway, significantly suppressing HCN emissions. Compared to traditional CuO catalysts, the CuO/Ti_1‐x_In_x_O_2_ catalyst achieves SCE of NO_x_ and CH_3_SH via coupling the reaction cycles of NO_x_ reduction and CH_3_SH oxidation with a lower reaction barrier. This study provides a new paradigm for achieving efficient and stable environmental catalysis via designing and constructing epitaxially structured oxide–oxide interface.

## Author Contributions

**Lupeng Han**: investigation, writing – original draft, visualization, validation, formal analysis, conceptualization. **Yanqing Li**: investigation, writing – original draft, methodology, visualization, formal analysis. **Yongjie Shen**: investigation, writing – original draft, methodology, formal analysis. **Huijun Yu**: investigation, writing – review and editing, visualization, formal analysis. **Evangelina Pensa**: writing – review and editing, data curation, validation. **Xuehui Yang**: methodology, writing – review and editing, formal analysis. **Yanqi Chen**: methodology, visualization, formal analysis. **Xiaonan Hu**: methodology, writing – review and editing. **Xiyang Wang**: methodology, writing – review and editing. **Song Li**: methodology, writing – review and editing, data curation. **Gaowu Qin**: data curation, supervision, resources, writing – review and editing. **Wenqiang Qu**: investigation, writing – review and editing. **Ming Xie**: methodology, writing – review and editing. **Emiliano Cortés**: funding acquisition, writing – review and editing, methodology, conceptualization, visualization, project administration, supervision, resources. **Dengsong Zhang**: conceptualization, investigation, funding acquisition, writing – original draft, data curation, supervision, resources, project administration.

## Conflicts of Interest

The authors declare no conflicts of interest.

## Supporting Information

## Supporting information



Experimental details, additional figures, and tables are compiled in the Supporting Information file. Additional references were cited within the Supporting Information.**Supporting File**: anie74225‐sup‐0001‐SuppMat.docx.

## Data Availability

The data that support the findings of this study are available from the corresponding author upon reasonable request.

## References

[1] C. H. Kim , G. Qi , K. Dahlberg , and W. Li , “Strontium‐Doped Perovskites Rival Platinum Catalysts for Treating NO_x_ in Simulated Diesel Exhaust,” Science 327 (2010): 1624–1627, 10.1126/science.1184087. 20339068

[2] J. Deng , X. Hu , A. J. Klaver , et al., “Catalyst Deactivation in the Abatement of Atmospheric Pollutants: Origin, Resistance, and Regeneration,” Chemical Reviews 125 (2025): 11260–11357, 10.1021/acs.chemrev.5c00426. 41289005

[3] D. Han , Z. Wang , H. Duan , et al., “Molecularly Engineered Co‐Phthalocyanine Covalent Organic Frameworks for Efficient Nitric Oxide‐to‐Ammonia Electroreduction,” Journal of the American Chemical Society 147 (2025): 41355–41366, 10.1021/jacs.5c10404. 41178038

[4] Y. Wang , T. Lan , L. Han , et al., “Non‐Precious Metal Catalysts with Gradient Oxidative Dual Sites Boost Bimolecular Activation for Catalytic Oxidation Reactions,” Angewandte Chemie International Edition 64 (2025): e202506018. 10.1002/anie.202506018 PMC12171384 40202179

[5] M. Elsener , R. J. G. Nuguid , O. Kröcher , and D. Ferri , “HCN Production From Formaldehyde during the Selective Catalytic Reduction of NO_x_ with NH_3_ over V_2_O_5_/WO_3_‐TiO_2_ ,” Applied Catalysis B: Environmental 281 (2021): 119462, 10.1016/j.apcatb.2020.119462.

[6] D. Zengel , P. Koch , B. Torkashvand , J. D. Grunwaldt , M. Casapu , and O. Deutschmann , “Emission of Toxic HCN during NO* _x_ * Removal by Ammonia SCR in the Exhaust of Lean‐Burn Natural Gas Engines,” Angewandte Chemie International Edition 59 (2020): 14423–14428, 10.1002/anie.202003670. PMC7497226 32391644

[7] F. Wang , T. Lan , X. Hu , et al., “Cl‐Resistant Synergistic Removal of NO_x_ and Chlorobenzene Over SmMn_2_O_5_‐Zeolite Composite Catalysts,” Environmental Science & Technology 59 (2025): 17361–17371, 10.1021/acs.est.5c06679. 40758917

[8] F. Wang , P. Wang , T. Lan , Y. Shen , W. Ren , and D. Zhang , “Ultralow‐Temperature NO_x_ Reduction Over SmMn_2_O_5_ Mullite Catalysts Via Modulating the Superficial Dual‐Functional Active Sites,” ACS Catalysis 12 (2022): 7622–7632, 10.1021/acscatal.2c01897.

[9] Y. Wen , S. Zhao , H. Yi , et al., “Efficient Catalytic Oxidation of Methyl Mercaptan to Sulfur Dioxide With NiCuFe Mixed Metal Oxides,” Environmental Technology & Innovation 26 (2022): 102252, 10.1016/j.eti.2021.102252.

[10] Q. Lian , L. Hu , D. Ma , et al., “Interstitial Atomic Bi Charge‐Alternating Processor Boosts Twofold Molecular Oxygen Activation Enabling Rapid Catalytic Oxidation Reactions at Room Temperature,” Advanced Functional Materials 32 (2022): 2205054, 10.1002/adfm.202205054.

[11] R. Zhang , H. Zhou , T. Shao , et al., “High‐Entropy Modulated High‐Spin Localized Cobalt Sites Enhance Catalytic Ozonation for Efficient Odor Control,” Angewandte Chemie International Edition 64 (2025): e202507109, 10.1002/anie.202507109. 40361285

[12] L. Han , S. Cai , M. Gao , et al., “Selective Catalytic Reduction of NO_x_ with NH_3_ by Using Novel Catalysts: State of the Art and Future Prospects,” Chemical Reviews 119 (2019): 10916–10976, 10.1021/acs.chemrev.9b00202. 31415159

[13] L. Ma , C. Y. Seo , M. Nahata , X. Chen , J. Li , and J. W. Schwank , “Shape Dependence and Sulfate Promotion of CeO_2_ for Selective Catalytic Reduction of NO with NH_3_ ,” Applied Catalysis B: Environmental 232 (2018): 246–259, 10.1016/j.apcatb.2018.03.065.

[14] S. Cheng , F. Xu , S. Yang , et al., “Modulating the Activity and SO_2_ Resistance of α‐Fe_2_O_3_ Catalysts for NH_3_–SCR of NOx via Crystal Facet Engineering,” Environmental Science & Technology 58 (2024): 8955–8965, 10.1021/acs.est.4c00276. 38718175

[15] A. B. Ngo , T. H. Vuong , H. Atia , et al., “Role of V and W Sites in V_2_O_5_−WO_3_/TiO_2_ Catalysts and Effect of Formaldehyde During NH_3_−SCR of NO_x_ ,” ChemCatChem 14 (2022): e202200175.

[16] A. B. Ngo , T. H. Vuong , H. Atia , et al., “Effect of Formaldehyde in Selective Catalytic Reduction of NO_x_ by Ammonia (NH_3_‐SCR) on a Commercial V_2_O_5_‐WO_3_/TiO_2_ Catalyst Under Model Conditions,” Environmental Science & Technology 54 (2020): 11753–11761, 10.1021/acs.est.0c00884. 32790302

[17] S. Barth , D. Zengel , T. J. Goncalves , et al., “Tailoring the Active Sites in Cu‐SSZ‐13 as a Catalyst for the Selective Catalytic Reduction of NH_3_ to Minimize HCHO and HCN Emissions,” Nature Catalysis 8 (2025): 804–821, 10.1038/s41929-025-01389-4.

[18] X. Han , K. Liu , M. Bian , et al., “SO_2_‐Induced Surface Defects on Monatomic Ce‐Doped MoTiO_x_ Catalyst for Efficient Reduction of NO_x_ With NH_3_ ,” Applied Catalysis B: Environment and Energy 366 (2025): 125021, 10.1016/j.apcatb.2025.125021.

[19] X. Fang , Y. Liu , Y. Cheng , and W. Cen , “Mechanism of Ce‐Modified Birnessite‐MnO_2_ in Promoting SO_2_ Poisoning Resistance for Low‐Temperature NH_3_‐SCR,” ACS Catalysis 11 (2021): 4125–4135, 10.1021/acscatal.0c05697.

[20] H. Pan , Z. Chen , X. Feng , et al., “Sulfur‐Resistant Mechanism of MnO_x_@CeO_2_@MgO Core–Shell Catalyst in Simultaneous Removal of NO and Chlorobenzene,” Applied Catalysis B: Environment and Energy 362 (2025): 124761, 10.1016/j.apcatb.2024.124761.

[21] L. Kang , L. Han , J. He , et al., “Improved NO_x_ Reduction in the Presence of SO_2_ by Using Fe_2_O_3_ ‐Promoted Halloysite‐Supported CeO_2_–WO_3_ Catalysts,” Environmental Science & Technology 53 (2019): 938–945, 10.1021/acs.est.8b05637. 30576117

[22] W. Zhao , Y. Tang , Y. Wan , et al., “Promotion Effects of SiO_2_ or/and Al_2_O_3_ Doped CeO_2_/TiO_2_ Catalysts for Selective Catalytic Reduction of NO by NH_3_ ,” Journal of Hazardous Materials 278 (2014): 350–359, 10.1016/j.jhazmat.2014.05.071. 24996153

[23] C. Liu , L. Chen , J. Li , et al., “Enhancement of Activity and Sulfur Resistance of CeO_2_ Supported on TiO_2_–SiO_2_ for the Selective Catalytic Reduction of NO by NH_3_ ,” Environmental Science & Technology 46 (2012): 6182–6189, 10.1021/es3001773. 22548347

[24] Z. Zheng , S. Wu , Z. Huang , H. Xu , and W. Shen , “Insights Into Enhancing SO_2_ Tolerance for Catalytic Combustion of Toluene over Sulfated CeZrO_x_ Supported Platinum Catalysts,” Colloids and Surfaces A: Physicochemical and Engineering Aspects 669 (2023): 131539, 10.1016/j.colsurfa.2023.131539.

[25] Z. Xie , K. Song , Y. Ren , et al., “Zr‐Doped TiO2–x Nano‐Oxide With Coordinatively Unsaturated Ti(Zr)–O Acid–Base Pairs for Efficient Propane Dehydrogenation,” ACS Catalysis 15 (2025): 6969–6978, 10.1021/acscatal.4c07810.

[26] Y. Yu , Z. Qin , X. Zhang , Y. Chen , G. Qin , and S. Li , “Far‐From‐equilibrium Processing Opens Kinetic Paths for Engineering Novel Materials by Breaking Thermodynamic Limits,” ACS Materials Letters 7 (2025): 319–332, 10.1021/acsmaterialslett.4c01952.

[27] H. Eskes , L. H. Tjeng , and G. A. Sawatzky , “Cluster‐Model Calculation of the Electronic Structure of CuO: A Model Material for the High‐Tc Superconductors,” Physical Review B 41 (1990): 288–299, 10.1103/PhysRevB.41.288. 9992767

[28] F. Wang , A. Chen , T. Lan , et al., “Synergistic Catalytic Removal of NO_x_ and Chlorinated Organics Through the Cooperation of Different Active Sites,” Journal of Hazardous Materials 468 (2024): 133722, 10.1016/j.jhazmat.2024.133722. 38367433

[29] P. Zhang , P. Wang , A. Chen , et al., “Alkali‐Resistant Catalytic Reduction of NO_x_ by Using Ce–O–B Alkali‐Capture Sites,” Environmental Science & Technology 55 (2021): 11970–11978, 10.1021/acs.est.1c02882. 34488354

[30] W. Hu , J. He , X. Liu , et al., “SO_2_ and H_2_O‐tolerant Catalytic Reduction of NO_x_ at a Low Temperature via Engineering Polymeric VO_x_ Species by CeO_2_ ,” Environmental Science & Technology 56 (2022): 5170–5178, 10.1021/acs.est.1c08715. 35369692

[31] H. Li , Y. Wang , C. Zhang , et al., “Insight Into the Strong Brönsted Acid Sites on Isolated WOx‐Modified Pt/Zirconium Phosphate for Glycerol Efficient Hydrodeoxygenation,” Applied Catalysis B: Environmental 325 (2023): 122342, 10.1016/j.apcatb.2022.122342.

[32] C. Sun , J. Zhu , Y. Lv , et al., “Dispersion, Reduction and Catalytic Performance of CuO Supported on ZrO_2_‐Doped TiO_2_ for no Removal by CO,” Applied Catalysis B: Environmental 103 (2011): 206–220, 10.1016/j.apcatb.2011.01.028.

[33] X. Wang , Z. Li , X. Li , et al., “Embedding Reverse Electron Transfer Between Stably Bare Cu Nanoparticles and Cation‐Vacancy CuWO_4_ ,” Advanced Materials 36 (2024): 2412570, 10.1002/adma.202412570. PMC11619222 39400389

[34] X. Song , W. Li , X. Liu , et al., “Oxygen Vacancies Enable the Visible Light Photoactivity of Chromium‐Implanted TiO_2_ Nanowires,” Journal of Energy Chemistry 55 (2021): 154–161, 10.1016/j.jechem.2020.07.013.

[35] D. An , J. Ji , Q. Cheng , et al., “Facile H_2_O‐Contributed O_2_ Activation Strategy over Mn‐Based SCR Catalysts to Counteract SO_2_ Poisoning,” Environmental Science & Technology 57 (2023): 14737–14746, 10.1021/acs.est.3c04314. 37738479

[36] X. Wang , Q. Yang , X. Li , et al., “Exploring the Dynamic Evolution of Lattice Oxygen on Exsolved‐Mn_2_O_3_@SmMn_2_O_5_ Interfaces for no Oxidation,” Nature Communications 15 (2024): 7613, 10.1038/s41467-024-51473-9. PMC11369115 39223132

[37] R. Tian , J. Lu , Z. Xu , et al., “Unraveling the Synergistic Reaction and the Deactivation Mechanism for the Catalytic Degradation of Double Components of Sulfur‐Containing VOCs Over ZSM‐5‐Based Materials,” Environmental Science & Technology 57 (2023): 1443–1455, 10.1021/acs.est.2c04033. 36196013

[38] G. Busca , J. Lamotte , J. C. Lavalley , and V. Lorenzelli , “FT‐IR Study of the Adsorption and Transformation of Formaldehyde on Oxide Surfaces,” Journal of the American Chemical Society 109 (1987): 5197–5202, 10.1021/ja00251a025.

[39] T. Di , B. Zhu , B. Cheng , J. Yu , and J. Xu , “A Direct Z‐Scheme G‐C_3_N_4_/SnS_2_ Photocatalyst With Superior Visible‐Light CO_2_ Reduction Performance,” Journal of Catalysis 352 (2017): 532–541, 10.1016/j.jcat.2017.06.006.

[40] X. Zu , Y. Zhao , X. Li , et al., “Ultrastable and Efficient Visible‐Light‐Driven CO_2_ Reduction Triggered by Regenerative Oxygen‐Vacancies in Bi_2_O_2_CO_3_ Nanosheets,” Angewandte Chemie International Edition 60 (2021): 13840–13846, 10.1002/anie.202101894. 33786954

[41] X. Liu , P. Wang , Y. Shen , S. Bi , W. Ren , and D. Zhang , “Boosting SO_2_‐Tolerant Catalytic Reduction of NO_x_ via Selective Adsorption and Activation of Reactants Over Ce^4+^–SO_4_ ^2–^ Pair Sites,” ACS Catalysis 12 (2022): 11306–11317, 10.1021/acscatal.2c02699.

[42] B. Jiang , Z. Wu , Y. Liu , S. C. Lee , and W. Ho , “DRIFT Study of the SO_2_ Effect on Low‐Temperature SCR Reaction Over Fe−Mn/TiO_2_ ,” Journal of Physical Chemistry C 114 (2010): 4961–4965, 10.1021/jp907783g.

[43] J. Wu , Z. Huang , Z. He , et al., “Unraveling the Role of CH_3_S* Intermediates for Efficient Methane and Hydrogen Sulfide Reforming Over Mo/Al_2_O_3_ Catalysts,” Journal of Catalysis 448 (2025): 116168, 10.1016/j.jcat.2025.116168.

[44] X. Li , X. Wang , Y. Wang , et al., “Hole‐Mediated Lattice Oxygen Redox Design for Perovskite Oxide Catalysts,” Angewandte Chemie International Edition 64 (2025): e202424347, 10.1002/anie.202424347. 39963987

[45] Q. Zhang , J. Fan , P. Ning , et al., “In Situ DRIFTS Investigation of NH_3_‐SCR Reaction over CeO_2_/Zirconium Phosphate Catalyst,” Applied Surface Science 435 (2018): 1037–1045, 10.1016/j.apsusc.2017.11.180.

[46] G. Yang , H. Zhao , X. Luo , et al., “Promotion Effect and Mechanism of the Addition of Mo on the Enhanced Low Temperature SCR of NO_x_ by NH_3_ over MnO_x_/γ‐Al_2_O_3_ Catalysts,” Applied Catalysis B: Environmental 245 (2019): 743–752, 10.1016/j.apcatb.2018.12.080.

[47] X. Weng , X. Dai , Q. Zeng , Y. Liu , and Z. Wu , “DRIFT Studies on Promotion Mechanism of H_3_PW_12_O_40_ in Selective Catalytic Reduction of NO With NH_3_ ,” Journal of Colloid and Interface Science 461 (2016): 9–14, 10.1016/j.jcis.2015.09.004. 26397902

[48] Y. Wang , Z. Liu , J. Li , P. An , and Z. Liu , “Unveiling the Promoting Effects of Zr and the Hydrothermal Treatment on the NH_3_‐SCR Activity of the Cu‐SAPO‐18 Catalyst,” Environmental Science & Technology 59 (2025): 2827–2837, 10.1021/acs.est.4c07935. 39883851

[49] V. M. Ludmila , E. A. Paukshtis , and G. I. Kazimira , “Nitration of Aromatics by Nitrogen Oxides on Zeolite Catalysts: Comparison of Reaction in the Gas Phase and Solutions,” Catalysis Reviews 37 (1995): 179–226, 10.1080/01614949508007095.

[50] L. Han , X. Wang , F. Wang , et al., “Environmental Catalysis for no_x_ Reduction by Manipulating the Dynamic Coordination Environment of Active Sites,” Environmental Science & Technology 59 (2025): 2306–2316, 10.1021/acs.est.4c11440. 39846481

