# Supplementary material for: Epitaxial Oxide Interfaces Create Poison‐Resistant CuO Sites for Environmental Catalysis
Source: Angew Chem Int Ed Engl. 2026 Aug 12;65(41):e7111765. doi: 10.1002/anie.7111765 (PMC13638093; doi:10.1002/anie.7111765)
Supplement: Supplementary file 1 [file ANIE-65-e7111765-s001.docx]

***Supporting Information***

**Epitaxial Oxide Interfaces Create Poison-Resistant CuO Sites for Environmental Catalysis**

Lupeng Han^a+^, Yanqing Li^a+^, Yongjie Shen^c+^, Huijun Yu^a^, Evangelina Pensa^b^, Xuehui Yang^a^, Yanqi Chen^a^, Xiaonan Hu^a^, Xiyang Wang^d^*, Song Li^d^, Gaowu Qin^d^, Wenqiang Qu^e^, Ming Xie^f^, Emiliano Cortés^b^* and Dengsong Zhang^a^*

[a]	Prof. L. Han, Y. Li, Dr. Y. Shen, H. Yu, X. Yang, Y. Chen, Dr. X. Hu, Dr. W. Qu, Prof. D. Zhang

International Joint Laboratory of Catalytic Chemistry, State Key Laboratory of Materials for Advanced Nuclear Energy, Innovation Institute of Carbon Neutrality, Department of Chemistry, College of Sciences

Shanghai University, Shanghai 200444, People’s Republic of China. E-mail: [dszhang@shu.edu.cn](mailto:dszhang@shu.edu.cn)

[b]	Dr. E. Pensa, Prof. E. Cortes

Nanoinstitute Munich, Faculty of Physics, Ludwig-Maximilians-Universität (LMU)

Munich 80539, Germany. E-mail: [Emiliano.Cortes@lmu.de](mailto:Emiliano.Cortes@lmu.de)

[c]	Dr. Y. Shen

Institute for Chemical Reaction Design and Discovery (WPI-ICReDD), Hokkaido University, Sapporo 001-0021, Japan.

[d]	Prof. X. Wang, Prof. S. Li, Prof. G. Qin

Key Laboratory for Anisotropy and Texture of Materials (Ministry of Education), School of Materials Science and Engineering, Northeastern University, Shenyang 110819, China. E-mail: [wangxiyang@mail.neu.edu.cn](mailto:wangxiyang@mail.neu.edu.cn)

[e]	Dr. W. Qu

Department of Chemistry, University of Toronto, 80 St. George Street, Toronto, ON M5S 3H6, Canada.

[f]	Dr. M. Xie

Department of Chemical Engineering, University of Bath, Bath, BA27AY UK.

[+]	These authors contributed equally to this work.

**Contents**

**1. Methods**

**2. Supporting Figures**

**Figure S1.** TEM-EDS mapping images of Ti_1-x_In_x_O_2_.

**Figure S2.** Raman spectra of CuO/TiO_2_ and CuO/Ti_1-x_In_x_O_2_.

**Figure S3.** (a) BF-STEM image of CuO/Ti_1-x_In_x_O_2_. (b) The intensity profile of (003) facet of TiO_2_. (c) The intensity profile of (200) facet of TiO_2_. (d) The intensity profile of (010) facet of CuO.

**Figure S4.** (a) HAADF-STEM image of CuO/Ti_1-x_In_x_O_2_. (b) The intensity profile of (006) facet of TiO_2_.

**Figure S5.** Ti K-edge FT-EXAFS spectra of CuO/TiO_2_ and CuO/Ti_1-x_In_x_O_2_ in R-space.

**Figure S6.** (a) HAADF-STEM image of CuO/Ti_1-x_In_x_O_2_. (b) The intensity profile of (100) facet of CuO. (c) The intensity profile of (001) facet of CuO.

**Figure S7.** (a) HAADF-STEM image of CuO/TiO_2_. (b) The intensity profile of (001) facet of CuO. (c) The intensity profile of (100) facet of CuO. (d) The image simulations of CuO in CuO/TiO_2_.

**Figure S8.** Experimental curves of EXAFS spectra for CuO, CuO/TiO_2_ and CuO/Ti_1-x_In_x_O_2_ in k-space.

**Figure S9.** Optimized model for CuO/TiO_2_ (a) and CuO/Ti_1-x_In_x_O_2_ (b).

**Figure S10.** The charge density difference and Bader charge analysis of CuO/TiO_2_ (a) and CuO/Ti_1-x_In_x_O_2_ (b). Yellow and cyan regions denote electron accumulation and depletion, respectively.

**Figure S11**. Py-IR profiles of TiO_2_, Ti_1-x_In_x_O_2_, CuO/TiO_2_, and CuO/Ti_1-x_In_x_O_2_ at 200 °C.

**Figure S12**. NH_3_-TPD-MS profiles (m/z =17) of TiO_2_, Ti_1-x_In_x_O_2_, CuO/TiO_2_ and CuO/Ti_1-x_In_x_O_2_.

**Figure S13**. O 1s XPS spectra of TiO_2_, Ti_1-x_In_x_O_2_, CuO/TiO_2_ and CuO/Ti_1-x_In_x_O_2_.

**Figure S14**. EPR spectra of TiO_2_, Ti_1-x_In_x_O_2_, CuO/TiO_2_ and CuO/Ti_1-x_In_x_O_2_.

**Figure S15**. The concentration of HCN (a) and CO (b) generated during the synergistic eliminating of NO_x_ and CH_3_SH as a function of reaction temperature over the CuO/TiO_2_ and CuO/Ti_1-x_In_x_O_2_. Reaction conditions: 500 ppm NO, 500 ppm NH_3_, 100 ppm CH_3_SH, 5 vol% O_2_, N_2_ as the balance gas, and GHSV of 25,000 h^-1^.

**Figure S16**. Plots of NO_x_ conversion and N_2_ selectivity as a function of reaction temperature over CuO/TiO_2_ and CuO/Ti_1-x_In_x_O_2_ in the absence of CH_3_SH. Reaction conditions: 500 ppm NO, 500 ppm NH_3_, 100 ppm CH_3_SH, 5 vol% O_2_, N_2_ as the balance gas, and GHSV of 25,000 h^-1^.

**Figure S17**. Arrhenius plot of CH_3_SH oxidation over CuO/TiO_2_ and CuO/Ti_1-x_In_x_O_2_.

**Figure S18**. Arrhenius plot of NH_3_-SCR over CuO/TiO_2_ and CuO/Ti_1-x_In_x_O_2_.

**Figure S19.** Plots of CH_3_SH conversion (a) and NO_x_ conversion (b) as a function of reaction time over CuO/TiO_2_ and CuO/Ti_1-x_In_x_O_2_ at the reaction conditions: 300 °C. 500 ppm NO, 500 ppm NH_3_, 100 ppm CH_3_SH, 5 vol% O_2_, N_2_ as the balance gas, and GHSV of 25,000 h^-1^.

**Figure S20.** NO+O_2_-TPD-MS (m/z = 30) profiles of CuO/TiO_2_ and CuO/Ti_1-x_In_x_O_2_.

**Figure S21.** CH_3_SH+O_2_-TPD profiles (m/z = 48) of CuO/TiO_2_ and CuO/Ti_1-x_In_x_O_2_.

**Figure S22.** NO adsorption energy on Cu sites of CuO/TiO_2_ and CuO/Ti_1-x_In_x_O_2_.

**Figure S23.** NH_3_ adsorption energy on Cu sites of CuO/TiO_2_ and CuO/Ti_1-x_In_x_O_2_.

**Figure S24.** CH_3_SH adsorption energy on Cu sites of CuO/TiO_2_ and CuO/Ti_1-x_In_x_O_2_.

**Figure S25.** *In situ* DRIFT spectra of the transient reactions between O_2_ and pre-adsorbed CH_3_SH at 240 ^o^C as a function of time over CuO/TiO_2_ and CuO/Ti_1-x_In_x_O_2_. Testing conditions: CH_3_SH (1000 ppm), O_2_ (5 vol %), N_2_ as the balance gas.

**Figure S26.** Cu 2p XPS spectra of CuO/TiO_2_ , CuO/Ti_1-x_In_x_O_2_, S-CuO/TiO_2_, and S-CuO/Ti_1-x_In_x_O_2_.

**Figure S27.** Cu K-edge XANES spectra of the CuO/TiO_2_ , CuO/Ti_1-x_In_x_O_2_, S-CuO/TiO_2_, and S- CuO/Ti_1-x_In_x_O_2_.

**Figure S28.** The R-space Cu K-edge EXAFS spectra of CuO/TiO_2_ , CuO/Ti_1-x_In_x_O_2_, S-CuO/TiO_2_, and S-CuO/Ti_1-x_In_x_O_2_.

**Figure S29.** The element line scanning diagram of HRTEM-EDS over S-CuO/TiO_2_ (a) and S-CuO/Ti_1-x_In_x_O_2_ (b).

**Figure S30.** In 3d XPS spectra of the CuO/Ti_1-x_In_x_O_2_ and S-CuO/Ti_1-x_In_x_O_2_.

**Figure S31**. The calculated Gibbs free energy diagrams over CuO/TiO_2_.

**Figure S32.** Relative energy diagram of *SH and *SO_2_ on Cu and In sites of CuO/Ti_1-x_In_x_O_2_, respectively.

**Figure S33.** The calculated relative energy diagrams of CH_3_SH oxidation and NH_3_-SCR over CuO/TiO_2_ and CuO/Ti_1-x_In_x_O_2_.

**3. Supporting Tables**

**Table S1.** Refined atomic positions, occupancies, and isotropic displacement parameters from XRD Rietveld refinement of CuO/Ti_1-x_In_x_O_2_.

**Table S2.** XRD Rietveld Refinement Results of CuO/Ti_1-x_In_x_O_2_ (Anatase).

**Table S3.** The FT-EXAFS fitting parameters of Ti K-edge for CuO/TiO_2_ and CuO/Ti_1-x_In_x_O_2_.

**Table S4.** The FT-EXAFS fitting parameters of Cu K-edge for CuCuO/TiO_2_ and CuO/Ti_1-x_In_x_O_2_.

**Table S5.** The quantitative analysis of Bader charge over CuO/TiO_2_ and CuO/Ti_1-x_In_x_O_2_.

**Table S6.** The amount of acid sites on TiO_2_, Ti_1-x_In_x_O_2_, Cu/TiO_2_ and CuO/Ti_1-x_In_x_O_2_ determined by NH_3_-TPD.

**Table S7.** The fitting parameters for the deactivation behavior of CuO/TiO_2_ and CuO/Ti_1-x_In_x_O_2_ obtained from the exponential decay model.

**Table S8.** Reaction energy diagram of each intermediate in the free energy calculations over CuO/Ti_1-x_In_x_O_2_.

**Table S9.** Reaction energy diagram of each intermediate in the free energy calculations over CuO/TiO_2_.

**Methods**

**1. Reagents and materials**

Cu(NO_3_)_2_·3H_2_O, tetrabutyl titanate, anhydrous ethanol, HNO_3_ were purchased from Sinopharm Chemical Reagent Co., Ltd. (China) without further purification. In(NO_3_)_3_·4.5H_2_O were purchased from Shanghai Macklin Biochemical Co., Ltd.

**2. Synthetic Procedures**

**2.1 Synthesis of TiO_2_ support**

9 mL tetrabutyl titanate was first mixed with 15 mL anhydrous ethanol to obtain a transparent solution and denoted as solution A. Solution B was then formulated by mixing 2 mL anhydrous ethanol, 2.8 mL deionized water, and 0.9 mL HNO_3_. Solution B was added dropwise to solution A under magnetic stirring at 25 °C. The sol was gelled and dried at 80 °C. Then the precursor was pulverized with agate mortar and calcined in a muffle furnace at 500 °C for 5 h with a ramping rate of 2 °C/min.

**2.2 Synthesis of** **Ti_1-x_In_x_O_2_ support**

9 mL tetrabutyl titanate was first mixed with 15 mL anhydrous ethanol to obtain a transparent solution and denoted as solution A. 0.915 g In(NO_3_)_3_·4.5H_2_O (the mole ratio of In/Ti is 1/9) was dissolved in 2 mL anhydrous ethanol, 2.8 mL deionized water and 0.9 mL HNO_3_, which was labeled as solution B. Solution B was added dropwise to solution A under magnetic stirring at 25°C. The sol was gelled and dried at 80 °C. Then the precursor was pulverized with agate mortar and calcined at 500 °C in air for 5 h with a ramping rate of 2 °C/min.

**2.3 Synthesis of CuO/TiO_2_ and CuO/Ti_1-x_In_x_O_2_**

CuO/TiO_2_ and CuO/Ti_1-x_In_x_O_2_ catalysts were prepared by an impregnation method. A designated amount of Cu(NO_3_)_2_·3H_2_O (CuO is loaded at 10 wt% on the support) was dissolved in 25 mL of deionized water. Then, the TiO_2_ and Ti_1-x_In_x_O_2_ powders were added to the aforementioned solution under continuous stirring for 1 h. Whereafter, the mixture was dried by the rotary evaporation at 55 °C. Finally, the dried mixture was calcined at 500 °C in air for 5 h with a ramping rate of 2 °C/min.

**3. Catalytic Performance Evaluation**

300 mg catalyst with 40–60 mesh was loaded into a 7 mm inner diameter quartz tube for NO_x_ and CH_3_SH conversion testing in a fixed–bed reactor. The gas composition was 500 ppm of NO, 500 ppm of NH_3_, 100 ppm of CH_3_SH, 5 vol % O_2_, and N_2_ as the balance gas. The concentration choices for NO and CH_3_SH were determined combining the literature and actual work condition in industries. The gas hourly space velocity (GHSV) was controlled at 25,000 h^-1^, corresponding to the total flow rate of 125 mL/min. The concentrations of NO, NO_2_, N_2_O, NH_3_, CH_3_SH, CO_2_, CO, HCN, and SO_2_ at the outlet of the reactor were measured by an atmosFIRt (AFS–B2T–C–2221). The GHSV was calculated by the following formula:

$$GHSV= \frac{q_{v}}{\pi hr^{2}}$$

where q_v_ represents the total flow rate, h stands for the height of the catalysts in the reactor, and r denotes the inner radius of the reactor. Record the catalytic performance of the catalysts when the reaction system is in a stable state with temperature decreases.

NO_x_ conversion and N_2_ selectivity were calculated by the following equations:

$$\mathrm{NO}_{x} conversion(\%)=\frac{{{[NO}_{x}]}_{\mathrm{in}}-{{[NO}_{x}]}_{\mathrm{out}}}{{{[NO}_{x}]}_{\mathrm{in}}}\times100\%$$

$$N_{2}\mathrm{selectivity}\left( \% \right)=\left( 1-\frac{{{2[N}_{2}O]}_{\mathrm{out}}}{{{[NO}_{x}]}_{\mathrm{in}}+{{[NH}_{3}]}_{\mathrm{in}}-{{[NO}_{x}]}_{\mathrm{out}}+{{[NH}_{3}]}_{\mathrm{out}}} \right)\times100\%$$

where NO_x_ denotes the total concentration of NO and NO_2_. [NO_x_]_in_, [NH_3_]_in_, [NO_x_]_out_, [N_2_O]_out_, and [NH_3_]_out_ denote the corresponding inlet and outlet gas concentrations, respectively.

CH_3_SH conversion and CO_2_ selectivity were calculated by the following equations:

$$\mathrm{CH}_{3}SH conversion(\%)=\frac{{[\mathrm{CH}_{3}SH]}_{\mathrm{in}}-{[\mathrm{CH}_{3}SH]}_{\mathrm{out}}}{{[\mathrm{CH}_{3}SH]}_{\mathrm{in}}}\times100\%$$

$$\mathrm{CO}_{2} conversion(\%)=\frac{{[\mathrm{CO}_{2}]}_{\mathrm{out}}}{{[\mathrm{CH}_{3}SH]}_{\mathrm{in}}-{[\mathrm{CH}_{3}SH]}_{\mathrm{out}}}\times100\%$$

where [CH_3_SH]_in_, [CH_3_SH]_out_, and [CO_2_]_out_ denote the corresponding inlet and outlet gas concentrations, respectively.

Kinetic experiments were conducted by the same procedure with NO_x_/CH_3_SH conversion restrained under 20% by varying the weight of catalyst. The apparent activation energies (E_a_ in kJ/mol) over the catalysts were determined following the Arrhenius equation.

**4. Characterization of the Catalysts**

XRD spectra recorded in the range of 20° to 80° were measured on a Rigaku D/MAS-RB X-ray diffractometer with Cu Kα (40 kV, 40 mA) radiation. Raman spectra in the range of 150–1500 cm^-1^ were recorded on the LabRAM HR Evolution using a laser with a wavelength of 532 nm and the spectral resolution employed was 4 cm^-1^. HAADF-STEM and BF-STEM were performed at aberration-corrected transmission electron microscope (JEM-ARM300F). The atomic-scale images were processed using Gatan Micrograph Suite software. TEM-EDS measurements were conducted on JEOL JEM-2010F. XPS was carried out on a PerkinElmer PHI-5300CESCA system with Mg Kα radiation. Adventitious carbon (C 1s = 284.8 eV) was used as a reference to calibrate binding energies of all elements. For H_2_-TPR, it was conducted on a Micromeritics AutoChem 2950 II auto-adsorption apparatus with a thermal conductivity detector (TCD). Before the process of reduction, 80 mg of each sample was treated under He atmosphere with a flow rate of 30 mL/min at 300 °C for 30 min, then cooled to 50 ^o^C under He atmosphere. In H_2_-TPR runs, catalysts were exposed to 10% H_2_/Ar and then the reactor temperature was raised from 50 °C to 800 °C with a rate of 10 °C/min. EPR spectra were recorded on JES X320 at a temperature of -196 °C. Py-IR were performed on Thermal Fisher 6700 spectrometer with a costumed vacuum transmission cell from Xiamen Tuosi Company. 0.05 g catalyst was pressed into tablet and degassed at 300 °C under vacuum for 30 min. Then the background was collected at different temperatures. The cell was sealed, and the vapor of pyridine was inlet to the cell for 10 min to reach pressure equilibrium at 30 °C. Then the cell was degassed under vacuum at 30 °C to remove excess pyridine. The spectrum was collected at 30 °C and 210 °C under vacuum. The Micromeritics AutoChem 2920 II auto-adsorption apparatus was employed for NH_3_-TPD-MS experiments with a TCD and Mass spectrometer (OMNISTAR) to monitor the NH_3_. Prior to NH_3_-TPD experiments, 80 mg of catalysts were outgassed under He protection (30 mL/min) at 300 °C for 30 min and then cooled to 100 °C. Samples were exposed to 10% NH_3_/He for adsorbing 1 h at 100 °C, the physical adsorption of ammonia was removed by He purging for 30 min at the same temperature. Finally, the temperature was raised to 800 °C with a ramping rate of 10 °C/min under He flow accompanied with the recording of the TCD signal and MS signal of NH_3_ at m/z = 17. The Micromeritics AutoChem 2920 II auto-adsorption apparatus was employed for CO_2_-TPD-MS experiments with a TCD and Mass spectrometer (OMNISTAR) to monitor the CO_2_. Prior to CO_2_-TPD experiments, 80 mg of catalysts were outgassed under He protection (30 mL/min) at 300 °C for 30 min and then cooled to 50 °C. Samples were exposed to 10% CO_2_/He for 1 h at 50 °C, the physical adsorption of ammonia was removed by He purging for 30 min at the same temperature. Finally, the temperature was raised to 800 °C with a ramping rate of 10 °C/min under He accompanied with the recording of the TCD signal and MS signal of CO_2_ at m/z = 44. CH_3_SH+O_2_-TPD-MS was carried out on a chemisorption analyzer (VODO, VDsorb-91i) equipped with a TCD detector and mass spectrometer (OMNISTARTM). First, 80 mg sample was pre-treated under a high purity He atmosphere with a flow rate of 30 mL/min for 30 min at 300 °C. After the sample was cooled to 50 °C under He, it was exposed to a flow of 1000 ppm CH_3_SH + 5 vol % O_2_ at the same temperature for 1 h. Then the temperature was raised to 800 °C at 10 °C/min under He accompanied with the recording of the TCD signal and MS signal of CH_3_SH at m/z = 48. NO+O_2_-TPD-MS was carried out on a chemisorption analyzer (VODO, VDsorb-91i) equipped with a TCD detector and mass spectrometer (OMNISTARTM). First, 80 mg sample was pre-treated under a high purity He atmosphere with a flow rate of 30 mL/min for 30 min at 300 °C. After the sample was cooled to 50 °C under He, it was exposed to a flow of 500 ppm NO + 5 vol % O_2_ at the same temperature for 1 h. Then the temperature was raised to 800 °C at 10 °C/min under He accompanied with the recording of the TCD signal and MS signal of NO at m/z = 30. Cu K-edge and Ti K-edge XAFS spectra are tested in the BL17B1 beamline of Shanghai Synchrotron Radiation Facility (SSRF). The XAFS spectra of these samples are all characterized at room temperature under the air atmosphere using Fluorescence mode. The data treatment and fitting were performed using the Athena and Artemis software packages. The Ti K-edge and Cu K-edge XANES spectra were normalized with Athena, and the corresponding k-space and R-space EXAFS Fourier transform functions were jointly fitted with Artemis, employing calculated CIF profiles to model the coordination structures. A k-weight of 3 was applied during the EXAFS fitting for both edges. The fitting ranges were as follows: for Ti K-edge EXAFS, k-range = 2.5-11.5 and R-range = 1-3.2 ; for Cu K-edge EXAFS, k-range = 2.5-11.8 and R-range = 1-3.5. Cu L-edge, Ti L-edge, O K-edge XANES spectra are measured at the beamline stations of the MCD-A and MCD-B (Soochow Beamline for Energy Materials) in national synchrotron radiation laboratory (NSRL). During the test process of the soft XANES spectrum, the vacuum degree in chamber is about 5×10-8 Pa and the test mode was the all-electron yield mode. The EXAFS raw data were background subtracted, normalized and Fourier transformed by standard procedures with the ATHENA program. The acquired EXAFS data were processed according to the standard procedures using the ATHENA module implemented in the IFEFFIT software packages. The k^3^-weighted EXAFS spectra were obtained by subtracting the post-edge background from the overall absorption and then normalizing with respect to the edge-jump step.

**5. *In-situ* DRIFT spectra measurements**

*In-situ* DRIFT measurements were performed on INVENIO R spectrometer equipped with a diffuse reflective *in situ* device. All *in situ* DRIFT were collected in the wavenumber range of 4000 to 1000 cm^-1^ in the absorbance format, accumulating 128 scans per minute at 4 cm^-1^ resolution. Prior to each test, each sample was pre-treated at 300 °C under N_2_ flow for 30 min and then regulated to the target temperature to obtain a background spectrum which should be deducted from the sample spectra.

For *in-situ* DRIFT spectra of CH_3_SH+O_2_ over the fresh catalyst, CH_3_SH+O_2_ flow were introduced to the cell at 240 °C for 30 min and the spectra were recorded with Macro software. Testing conditions: CH_3_SH (1000 ppm), O_2_ (5 vol %), N_2_ as the balance gas.

For *in-situ* DRIFT spectra of transient reactions between CH_3_SH and O_2_ over the fresh catalyst, CH_3_SH flow was first introduced to the cell at 240 °C for 30 min. Next, a N_2_ flow was applied for 10 minutes to purge catalyst. Then, O_2_ was introduced to the cell to initiate titration reactions under continuous IR scanning and recorded with Macro software. Testing conditions: CH_3_SH (1000 ppm), O_2_ (5 vol %), N_2_ as the balance gas.

For *in-situ* DRIFT spectra of transient reactions between NH_3_ (or NO+O_2_) and NO+O_2_ (or NH_3_) over the pre-treated catalysts by CH_3_SH+O_2_, CH_3_SH+O_2_ flow were first introduced to the cell at 240 °C for 30 min. Next, a N_2_ flow was applied for 10 minutes to purge catalyst. Prior to the following test, each sample pre-treated by CH_3_SH+O_2_ was regulated to the target temperature to obtain a background spectrum under N_2_ flow which should be deducted from the sample spectra once again. Subsequently, NO+O_2_ (or NH_3_) was introduced to the cell at 240 °C for 30 min. Next, a N_2_ flow was applied for 10 minutes to purge catalyst. Then, NH_3_ (or NO+O_2_) was introduced to the cell to initiate titration reactions under continuous IR scanning and recorded with Macro software. Testing conditions: NO (1000 ppm), NH_3_ (1000 ppm), CH_3_SH (1000 ppm), O_2_ (5 vol %), N_2_ as the balance gas.

**6. Details of** **DFT Calculations**

The DFT calculations were performed under periodic boundary conditions by using the Vienna Ab initio Simulation Package (VASP) version 6^[1]^. The PAW method^[2]^ was adopted for calculations with spin polarization and van der Waals corrections (DFT-D3)^[3]^. The Perdew-Burke-Ernzerhof (PBE) exchange-correlation functional was used for calculations within the generalized gradient approximation (GGA)^[4]^. A (1 × 1 × 1) gamma mesh and (3 × 3 × 1) *k*-point grid was adopted for Brillouin zone sampling in structural optimization and electronic calculation, respectively. The cutoff energy was set to 500 eV. The DFT + U was applied to address the strong on-site Coulomb interaction with the U-J parameters of 4.2 and 6.0 eV for Ti 3d and Cu 3d^[5]^. Electronic energies and residual forces converged to 10^-5^ eV and -0.02 eV·Å^-1^, respectively. The crystal structures of bulk rutile and anatase TiO_2_ were obtained from the Materials Project database^[6]^. The rutile TiO_2_ (110) and anatase TiO_2_ (101) surface, which both consists of three layers, was used for the slab model, and the bottom one layer was fixed. The charge transfer was obtained by Bader calculation. Charge density differences were processed by VASPKIT and visualized by VESTA software^[7]^.

The adsorption energies (E_ads_) of the species (NH_3_, NO, O_2_, SO_2_, SO_4_ and other intermediates) were calculated by the following formula.

E_ads_ = E_species/surface_ – E_surface_ – E_species_	(1)

where E_species/surface_, E_surface_ and E_species_ represent the energies of the catalyst surface adsorbed species, the catalyst surface and the species, respectively.

The formation energies of the oxygen vacancy (E_Ov_) were calculated by the following formula.

E_Ov_ = E_surface_Ov_ – E_surface_ + 1/2E_O2_	(2)

where E_surface_Ov_ and E_O2_ represent the energies of the catalyst surface with one oxygen vacancy and the isolated O_2_ molecule.

Figure S1**.** TEM-EDS mapping images of Ti_1-x_In_x_O_2_.

The In elements are highly dispersed with Ti elements in the EDS mapping images of Ti_1-x_In_x_O_2_, which indicates the formation of Ti_1-x_In_x_O_2_ solid solutions.

Figure S2. Raman spectra of CuO/TiO_2_ and CuO/Ti_1-x_In_x_O_2_.

The Raman spectra of CuO/TiO_2_ and CuO/Ti_1-x_In_x_O_2_ show modes of anatase TiO_2_ with symmetries E_g_, B_1g_, A_1g_ and E_g_ observed at 144, 399, 516, and 639 cm^-1^, respectively. No other vibration modes found in CuO/Ti_1-x_In_x_O_2_ indicate the formation of Ti_1-x_In_x_O_2_ solid solutions.

Figure S3**.** (a) BF-STEM image of CuO/Ti_1-x_In_x_O_2_. (b) The intensity profile of (003) facet of TiO_2_. (c) The intensity profile of (200) facet of TiO_2_. (d) The intensity profile of (010) facet of CuO.

Figure S4**.** (a) HAADF-STEM image of CuO/Ti_1-x_In_x_O_2_. (b) The intensity profile of (006) facet of TiO_2_.

Figure S5**.** Ti K-edge FT-EXAFS spectra of CuO/TiO_2_ and CuO/Ti_1-x_In_x_O_2_ in R-space.

**Figure S6.** (a) HAADF-STEM image of CuO/Ti_1-x_In_x_O_2_. (b) The intensity profile of (100) facet of CuO. (c) The intensity profile of (001) facet of CuO.

**Figure S7.** (a) HAADF-STEM image of CuO/TiO_2_. (b) The intensity profile of (001) facet of CuO. (c) The intensity profile of (100) facet of CuO. (d) The image simulations of CuO in CuO/TiO_2_.

Figure S8**.** Experimental curves of EXAFS spectra for CuO, CuO/TiO_2_ and CuO/Ti_1-x_In_x_O_2_ in k-space.

Ti

O

Cu

In

Figure S9**.** Optimized model for CuO/TiO_2_ (a) and CuO/Ti_1-x_In_x_O_2_ (b).

Based on the XRD results, CuO/TiO_2_ is modeled by TiO_2_ (110) supported Cu_6_O_6_ (111) clusters while CuO/Ti_1-x_In_x_O_2_ is modeled by TiO_2_ (101) supported Cu_6_O_6_ (111) clusters.

Ti

O

Cu

In

Figure S10**.** The charge density difference and Bader charge analysis of CuO/TiO_2_ (a) and CuO/Ti_1-x_In_x_O_2_ (b). Yellow and cyan regions denote electron accumulation and depletion, respectively.

Figure S11. Py-IR profiles of TiO_2_, Ti_1-x_In_x_O_2_, CuO/TiO_2_, and CuO/Ti_1-x_In_x_O_2_ at 200 °C.

Figure S12. NH_3_-TPD-MS profiles (m/z =17) of TiO_2_, Ti_1-x_In_x_O_2_, CuO/TiO_2_ and CuO/Ti_1-x_In_x_O_2_.

Cu/TiInO_x_ shows one large NH_3_ desorption peak at a medium temperature (366 ^o^C), which is mainly attributed to the medium strong acid sites for the Lewis acidic CuO_x_ species. Besides, Cu/TiInO_x_ shows one weak NH_3_ desorption peak at a relatively high temperature (511 ^o^C), further demonstrating the contribution of Lewis acidic CuO_x_ species.

Figure S13. O 1s XPS spectra of TiO_2_, Ti_1-x_In_x_O_2_, CuO/TiO_2_ and CuO/Ti_1-x_In_x_O_2_.

Figure S14. EPR spectra of TiO_2_, Ti_1-x_In_x_O_2_, CuO/TiO_2_ and CuO/Ti_1-x_In_x_O_2_.

Figure S15. The concentration of HCN (a) and CO (b) generated during the synergistic eliminating of NO_x_ and CH_3_SH as a function of reaction temperature over the CuO/TiO_2_ and CuO/Ti_1-x_In_x_O_2_. Reaction conditions: 500 ppm NO, 500 ppm NH_3_, 100 ppm CH_3_SH, 5 vol% O_2_, N_2_ as the balance gas, and GHSV of 25,000 h^-1^.

Figure S16. Plots of NO_x_ conversion and N_2_ selectivity as a function of reaction temperature over CuO/TiO_2_ and CuO/Ti_1-x_In_x_O_2_ in the absence of CH_3_SH. Reaction conditions: 500 ppm NO, 500 ppm NH_3_, 100 ppm CH_3_SH, 5 vol% O_2_, N_2_ as the balance gas, and GHSV of 25,000 h^-1^.

Figure S17. Arrhenius plot of CH_3_SH oxidation over CuO/TiO_2_ and CuO/Ti_1-x_In_x_O_2_.

Figure S18. Arrhenius plot of NH_3_-SCR over CuO/TiO_2_ and CuO/Ti_1-x_In_x_O_2_.

Figure S19. Plots of CH_3_SH conversion (a) and NO_x_ conversion (b) as a function of reaction time over CuO/TiO_2_ and CuO/Ti_1-x_In_x_O_2_ at the reaction conditions: 300 °C. 500 ppm NO, 500 ppm NH_3_, 100 ppm CH_3_SH, 5 vol% O_2_, N_2_ as the balance gas, and GHSV of 25,000 h^-1^.

Figure S20**.** NO+O_2_-TPD-MS (m/z = 30) profiles of CuO/TiO_2_ and CuO/Ti_1-x_In_x_O_2_.

Figure S21**.** CH_3_SH+O_2_-TPD profiles (m/z = 48) of CuO/TiO_2_ and CuO/Ti_1-x_In_x_O_2_.

Figure S22**.** NO adsorption energy on Cu sites of CuO/TiO_2_ and CuO/Ti_1-x_In_x_O_2_.

Figure S23**.** NH_3_ adsorption energy on Cu sites of CuO/TiO_2_ and CuO/Ti_1-x_In_x_O_2_.

Figure S24**.** CH_3_SH adsorption energy on Cu sites of CuO/TiO_2_ and CuO/Ti_1-x_In_x_O_2_.

Figure S25**.** *In situ* DRIFT spectra of the transient reactions between O_2_ and pre-adsorbed CH_3_SH at 240 ^o^C as a function of time over CuO/TiO_2_ and CuO/Ti_1-x_In_x_O_2_. Testing conditions: CH_3_SH (1000 ppm), O_2_ (5 vol %), N_2_ as the balance gas.

Figure S26**.** Cu 2p XPS spectra of CuO/TiO_2_ , CuO/Ti_1-x_In_x_O_2_, S-CuO/TiO_2_, and S-CuO/Ti_1-x_In_x_O_2_.

Figure S27**.** Cu K-edge XANES spectra of the CuO/TiO_2_ , CuO/Ti_1-x_In_x_O_2_, S-CuO/TiO_2_, and S- CuO/Ti_1-x_In_x_O_2_.

Figure S28**.** The R-space Cu K-edge EXAFS spectra of CuO/TiO_2_ , CuO/Ti_1-x_In_x_O_2_, S-CuO/TiO_2_, and S-CuO/Ti_1-x_In_x_O_2_.

Figure S29**.** The element line scanning diagram of HRTEM-EDS over S-CuO/TiO_2_ (a) and S-CuO/Ti_1-x_In_x_O_2_ (b).

Figure S30**.** In 3d XPS spectra of the CuO/Ti_1-x_In_x_O_2_ and S-CuO/Ti_1-x_In_x_O_2_.

**Figure S31**. The calculated Gibbs free energy diagrams over CuO/TiO_2_.

Figure S32**.** Relative energy diagram of *SH and *SO_2_ on Cu and In sites of CuO/Ti_1-x_In_x_O_2_, respectively.

Figure S33**.** The calculated relative energy diagrams of CH_3_SH oxidation and NH_3_-SCR over CuO/TiO_2_ and CuO/Ti_1-x_In_x_O_2_.

Table S1. Refined atomic positions, occupancies, and isotropic displacement parameters from XRD Rietveld refinement of CuO/Ti_1-x_In_x_O_2_.

| **Site** | **x** | **y** | **z** | **Atom** | **Occupy** | **Beq** |
| --- | --- | --- | --- | --- | --- | --- |
| In | 0.00000 | 0.00000 | 0.00000 | In | 0.03132 | 0.002039 |
| Ti | 0.00000 | 0.00000 | 0.00000 | Ti | 0.9687 | 1.428 |
| O | 0.00000 | 0.00000 | 0.20559 | O | 0.9843 | 0.6999 |

|  | **Use** | **Value** | **Code** | **Error** |
| --- | --- | --- | --- | --- |
| **Use phase** | 1 |  |  |  |
| **Spacegroup** |  | I4₁/amd |  |  |
| **a (Å)** |  | **3.77856 (standard 3.77600)** | a_9015929 | 0 |
| **c (Å)** |  | **9.52405 (standard 9.48600)** | c_9015929 | 0 |
| **Scale** | 1 | 0.00671 | sc_9015929 | 0 |
| **Wt% rietveld** |  | 100 |  | 0 |
| **Wt% of spiked** | 0 | 0 |  |  |
| **Cell mass** |  | 325.847 |  | 0 |
| **Cell volume (Å^3)** |  | **135.97949 (standard 135.2531Å)** |  | 0 |
| **Cry LAC (1/cm)** |  | 511.151 |  | 0 |
| **Cry density (g/cm^3)** |  | 3.979 |  | 0 |
| **R bragg** |  | 1.577 |  |  |

Table S2. XRD Rietveld Refinement Results of CuO/Ti_1-x_In_x_O_2_ (Anatase).

**Table S3.** The FT-EXAFS fitting parameters of Ti K-edge for CuO/TiO_2_ and CuO/Ti_1-x_In_x_O_2_.

| **Sample** | **Shell** | **R (Å)** | **CN** | **ΔE_0_(eV)** | **σ^2^ (x10****^-3^ Å^2^)** | **R-factor** |
| --- | --- | --- | --- | --- | --- | --- |
| Ti foil | Ti-Ti | 2.86±0.03 | 6 | 4.1±0.5 | 4.8±0.6 | 0.018 |
|  | Ti-Ti | 2.93±0.02 | 6 | 4.1±0.5 | 4.8±0.6 |  |
| CuO/TiO_2_ | Ti-O | 1.94±0.03 | 5.7±0.3 | 4.4±0.8 | 4.8±0.4 | 0.017 |
|  | Ti-Ti/Cu | 2.83±0.03 | 1.8±0.5 | 4.4±0.8 | 4.8±0.6 |  |
| CuO/Ti_1-x_In_x_O_2_ | Ti-O | 1.98±0.02 | 5.4±0.3 | 1.5±0.6 | 1.5±0.6 | 0.019 |
|  | Ti-Ti/In/Cu | 2.90±0.03 | 1.9±0.6 | 1.5±0.6 | 1.5±0.6 |  |

CN is the coordination number; R is the distance between absorber and backscatter atoms; σ^2^ is the Debye-Waller factor to account for both thermal and structural disorders; ΔE_0_ is the inner potential correction (edge-energy shift); S02 fitting parameter is 0.7.

Table S4. The FT-EXAFS fitting parameters of Cu K-edge for CuCuO/TiO_2_ and CuO/Ti_1-x_In_x_O_2_.

| **Sample** | **Shell** | **R (Å)** | **CN** | **ΔE_0_(eV)** | **σ^2^ (x10^-3^ Å^2^)** | **R-factor** |
| --- | --- | --- | --- | --- | --- | --- |
| Cu foil | Cu-Cu | 2.55±0.03 | 12* | 3.4±0.5 | 3.8±0.5 | 0.011 |
| CuO | Cu-O | 1.96±0.02 | 4 | 1.1±0.3 | 4.4±0.5 | 0.018 |
|  | Cu-Cu (O) | 2.91±0.04 | 8 | 1.8±0.6 | 7.6±0.6 |  |
| CuO/TiO_2_ | Cu-O | 1.96±0.02 | 3.89±0.03 | 0.9±0.6 | 3.8±0.6 | 0.013 |
|  | Cu-Cu (O) | 2.90±0.04 | 6.8±0.6 | 5.7±0.9 | 5.6±0.8 |  |
| CuO/Ti_1-x_In_x_O_2_ | Cu-O | 1.94±0.02 | 3.99±0.02 | 2.8±0.4 | 4.1±0.4 | 0.015 |
|  | Cu-Cu (O) | 2.96±0.04 | 6.1±0.7 | 2.8±0.4 | 9.1±0.8 |  |

CN is the coordination number; R is the distance between absorber and backscatter atoms; σ^2^ is the Debye-Waller factor to account for both thermal and structural disorders; ΔE_0_ is the inner potential correction (edge-energy shift); S02 fitting parameter is 0.7.

Table S5. The quantitative analysis of Bader charge over CuO/TiO_2_ and CuO/Ti_1-x_In_x_O_2_.

| **Sample** | **All Cu atoms (e)** | **Per Cu atom (e)** |
| --- | --- | --- |
| Cu/TiO_2_ | -4.01 | -0.67 |
| CuO/Ti_1-x_In_x_O_2_ | -5.63 | -0.94 |

**Table** S6. The amount of acid sites on TiO_2_, Ti_1-x_In_x_O_2_, Cu/TiO_2_ and CuO/Ti_1-x_In_x_O_2_ determined by NH_3_-TPD.

| **Sample** | **Acid amount (μmol·g⁻¹)** | | | |
| --- | --- | --- | --- | --- |
|  | **＜250 ^o^C** | **250-500 ^o^C** | **＞500 ^o^C** | **Total** |
| TiO_2_ | - | 5.53 | - | 5.53 |
| Ti_1-x_In_x_O_2_ | 2.79 | 5.93 | - | 8.72 |
| Cu/TiO_2_ | 4.73 | 2.52 | - | 7.25 |
| CuO/Ti_1-x_In_x_O_2_ | 3.29 | 12.51 | 1.35 | 17.15 |

**Table S7.** The fitting parameters for the deactivation behavior of CuO/TiO_2_ and CuO/Ti_1-x_In_x_O_2_ obtained from the exponential decay model.

| Sample  Fitting data | CuO/TiO_2_  NO_x_ conversion | CuO/TiO_2_  CH_3_SH conversion | CuO/Ti_1-x_In_x_O_2_  NO_x_ conversion | CuO/Ti_1-x_In_x_O_2_  CH_3_SH conversion |
| --- | --- | --- | --- | --- |
| y_0_ | 0.71±0.08 | 7.20±0.61 | 5.76±0.09 | 33.20±0.34 |
| A | 7.45±0.22 | 38.15±1.21 | 6.63±0.18 | 12.99±0.73 |
| K_d_ | 1.35±0.08 | 0.84±0.06 | 0.41±0.03 | 0.43±0.06 |
| R^2^ | 0.98 | 0.98 | 0.97 | 0.90 |

The exponential decay model is expressed as: y = A·$\text{e}^{\text{-}\text{K}_{\text{d}}\text{·x}}$+ y_0_, where y represents the conversion, A is the initial amplitude, K_d_ is the deactivation rate constant, x is the time, and y_0_ is the asymptotic conversion.

Table S8. Reaction energy diagram of each intermediate in the free energy calculations over CuO/Ti_1-x_In_x_O_2_.

| **Structure** | **Reaction** | **ΔE/eV** |
| --- | --- | --- |
| 1. Catalyst(*) | * | 0.00 |
| 2. CH_3_SH* | * + CH_3_SH → CH_3_SH* | -1.18 |
| 3. CH_2_SH* | CH_3_SH* + 1/4O_2_ → CH_2_SH* + 1/2H_2_O | -1.06 |
| 4. H_2_CO* + SH*^(Cu)^ | CH_2_SH* + 1/2O_2_ + *(Cu) → H_2_CO* + SH*^(Cu)^ | -2.98 |
| 4.1 H_2_CO* + SH*^(In)^ | CH_2_SH* + 1/2O_2_ + *(In) → H_2_CO* + SH*^(In)^ | -2.29 |
| 5. HCOO* + SO_2_*^(In)^ | H_2_CO*+ SH*^(Cu)^ + 2O_2_ → HCOO* + SO_2_*^(In)^ + H_2_O | -10.23 |
| 5.1 HCOO* + SO_2_*^(Cu)^ | H_2_CO*+ SH*^(Cu)^ + 2O_2_ → HCOO* + SO_2_*^(Cu)^ + H_2_O | -9.90 |
| 6. CO_3_* + SO_4_*^(In)^ + H* | HCOO* + SO_2_*^(In)^ + * + 3/2O_2_ → CO_3_* + SO_4_*^(In)^ + H* | -11.79 |
| 7. SO_4_*^(In)^ + O* + H* | CO_3_* + SO_4_*^(In)^ + H* → SO_4_*^(In)^ + CO_2_ + O* + H* | -13.56 |
| 8. SO_4_*^(In)^ + NH_3_*^(Cu)^ + O* + H* | SO_4_*^(In)^ + NH_3_ + *(Cu) + O* + H* → SO_4_*^(In)^ + NH_3_*^(Cu)^ + O* + H* | -14.65 |
| 9. SO_4_*^(In)^ + NH_2_*^(Cu)^ | SO_4_*^(In)^ + NH_3_*^(Cu)^ + O* + H* → SO_4_*^(In)^ + NH_2_*^(Cu)^ + H_2_O | -14.34 |
| 10. SO_4_*^(In)^ + NH_2_NO*^(Cu)^ | SO_4_*^(In)^ + NH_2_*^(Cu)^ + NO → SO_4_*^(In)^ + NH_2_NO*^(Cu)^ | -15.74 |
| 11. SO_4_*^(In)^ | SO_4_*^(In)^ + NH_2_NO*(^Cu)^ → SO_4_^*(In)^ + * + N_2_ + H_2_O | -16.93 |

Table S9. Reaction energy diagram of each intermediate in the free energy calculations over CuO/TiO_2_.

| **Structure** | **Reaction** | **ΔE/eV** |
| --- | --- | --- |
| 1. Catalyst(*) | * | 0.00 |
| 2. CH_3_SH* | * + CH_3_SH → CH_3_SH* | -1.00 |
| 3. CH_2_SH* | CH_3_SH* + 1/4O_2_ → CH_2_SH* + 1/2H_2_O | -0.43 |
| 4. H_2_CO* + SH* | CH_2_SH* + 1/2O_2_ + *→ H_2_CO* + SH* | -2.20 |
| 5. HCOO* + SO_2_* | H_2_CO*+ SH* + 2O_2_ → HCOO* + SO_2_* + H_2_O | -10.60 |
| 6. CO_3_* + SO_4_* + H* | HCOO* + SO_2_* + * + 3/2O_2_ → CO_3_* + SO_4_* + H* | -13.73 |
| 7. SO_4_* + O* + H* | CO_3_* + SO_4_* + H* → SO_4_* + CO_2_ + O* + H* | -15.23 |
| 8. SO_4_* + NH_3_* + O* + H* | SO_4_* + NH_3_ + O* + * + H* → SO_4_* + NH_3_* + O* + H* | -15.84 |
| 9. SO_4_* + NH_2_* | SO_4_* + NH_3_* + O^*^ + H* → SO_4_* + NH_2_* + H_2_O | -14.95 |
| 10. SO_4_* + NH_2_NO* | SO_4_* + NH_2_* + NO → SO_4_* + NH_2_NO* | -16.03 |
| 11. SO_4_* | SO_4_* + NH_2_NO* → SO_4_* + * + N_2_ + H_2_O | -17.29 |

**References:**

[1]	a) G. Kresse, J. Furthmüller, *Computational Materials Science* **1996**, 6, 15-50; b) G. Kresse, J. Hafner, *Physical Review B* **1993**, 47, 558-561.

[2]	a) G. Kresse, D. Joubert, *Physical Review B* **1999**, 59, 1758-1775; b) O. Bengone, M. Alouani, P. Blöchl, J. Hugel, *Physical Review B* **2000**, 62, 16392-16401; c) P. E. Blöchl, *Physical Review B* **1994**, 50, 17953-17979.

[3]	S. Grimme, J. Antony, S. Ehrlich, H. Krieg, *The Journal of Chemical Physics* **2010**, 132, 154104.

[4]	J. P. Perdew, K. Burke, M. Ernzerhof, *Physical Review Letters* **1996**, 77, 3865-3868.

[5]	a) R. Qi, B. Zhu, Z. Han, Y. Gao, *ACS Catalysis* **2022**, 12, 8269-8278; b) Y. Shen, W. Kaewraung, M. Gao, *Physical Chemistry Chemical Physics* **2025**, 27, 5868-5875.

[6]	A. Jain, S. P. Ong, G. Hautier, W. Chen, W. D. Richards, S. Dacek, S. Cholia, D. Gunter, D. Skinner, G. Ceder, K. A. Persson, *APL Materials* **2013**, 1, 011002.

[7]	a) N. X. Vei Wang, Jin Cheng Liu, Gang Tang, Wen-Tong Geng, *Computer Physics Communications* **2021**, 267; b) K. M. F. Izumia, *Journal of Applied Crystallography* **2011**, 44, 1272-1276.
